# Consequences of combining siRNA-mediated DNA methyltransferase 1 depletion with 5-aza-2′-deoxycytidine in human leukemic KG1 cells

**DOI:** 10.18632/oncotarget.3317

**Published:** 2015-03-20

**Authors:** Stéphane Vispé, Arthur Deroide, Emeline Davoine, Cécile Desjobert, Fabrice Lestienne, Lucie Fournier, Natacha Novosad, Sophie Bréand, Jérôme Besse, Florence Busato, Jörg Tost, Luc De Vries, Didier Cussac, Joëlle Riond, Paola B. Arimondo

**Affiliations:** ^1^ Unité de Service et de Recherche n°3388 CNRS-Pierre Fabre, ETaC Epigenetic Targeting of Cancer, CRDPF, Toulouse, France; ^2^ Molecular and Cellular Biology Department, Centre de Recherche Pierre Fabre, Castres, France; ^3^ Informatique de Recherche (Bioinformatics and Statistics), Centre de Recherche Pierre Fabre, Castres, France; ^4^ Laboratory for Epigenetics and Environment, Centre National de Génotypage, CEA-Institut de Génomique, Evry, France

**Keywords:** leukemia, DNA methylation, DNMT, 5-aza-2′-deoxycytidine, DNA damage

## Abstract

5-azacytidine and 5-aza-2′-deoxycytidine are clinically used to treat patients with blood neoplasia. Their antileukemic property is mediated by the trapping and the subsequent degradation of a family of proteins, the DNA methyltransferases (DNMT1, DNMT3A, and DNMT3B) leading to DNA demethylation, tumor suppressor gene re-expression and DNA damage. Here we studied the respective role of each DNMT in the human leukemia KG1 cell line using a RNA interference approach. In addition we addressed the role of DNA damage formation in DNA demethylation by 5-aza-2′-deoxycytidine. Our data show that DNMT1 is the main DNMT involved in DNA methylation maintenance in KG1 cells and in mediating DNA damage formation upon exposure to 5-aza-2′-deoxycytidine. Moreover, KG1 cells express the DNMT1 protein at a level above the one required to ensure DNA methylation maintenance, and we identified a threshold for DNMT1 depletion that needs to be exceeded to achieve DNA demethylation. Most interestingly, by combining *DNMT1* siRNA and treatment with low dose of 5-aza-2′-deoxycytidine, it is possible to uncouple DNA damage formation from DNA demethylation. This work strongly suggests that a direct pharmacological inhibition of DNMT1, unlike the use of 5-aza-2′-deoxycytidine, should lead to tumor suppressor gene hypomethylation and re-expression without inducing major DNA damage in leukemia.

## INTRODUCTION

In recent years, epigenetic modifications in cancer have been largely investigated and have successfully led to the development of novel anticancer therapies aimed at reversing aberrant modification patterns, among which DNA methylation at cytosines in CpG dinucleotides is probably the best studied [1, 2]. In normal cells, these CpG are generally methylated, protecting the cells from DNA rearrangement, while in the promoters of tumor suppressor genes (TSG) the CpG grouped in structures called CpG islands (CGIs) are mostly unmethylated. But in many cancer cells, bulk DNA becomes largely unmethylated, allowing for the re-expression of embedded sequences associated with genomic instability. At the same time promoters of many TSG become hypermethylated, associated with the silencing of the corresponding genes [3]. Such deregulation has been used to identify and develop DNA methylation inhibitors, among which two “epidrugs” have been approved to treat myelodysplasic syndromes (MDS), Acute Myeloid Leukemia (AML) and Chronic MyeloMonocytic Leukemia (CMML), namely 5-aza-2′-deoxycytidine (Dacogen^®^, hereafter named DAC) and 5-aza-2′cytidine (Vidaza™) [4–7]. These cytosine analogs target a family of proteins, called DNA Methyltransferases or DNMTs, responsible for the transfer of a methyl group onto a cytosine in the context of a CpG dinucleotide. But their chemical instability translates into poor pharmacokinetics properties in human, and a more stable analogue, *i.e*. SGI-110, is presently being evaluated in clinical trials, both for blood and solid tumors [8–10]. Regarding their molecular mechanism of action, these nucleoside analogs incorporate into DNA, act as cytosine decoy and trap irreversibly the various DNMTs, inducing their proteasome-dependent degradation [11–14]. This loss of the DNMT enzymes then leads to passive demethylation of silenced TSGs associated with their re-expression, ultimately leading to cell growth arrest and cell death. Moreover, the formation of DNMT adducts in DAC-enriched DNA was shown to generate DNA damage in various solid cancer cell lines, and particularly DNA breaks inducing γH2AX phosphorylation [15–18]. Such lesions can contribute to active DNA demethylation through excision of methylated cytosines (mC) from the DNA, which are subsequently repaired and replaced by unmethylated cytosines, in addition to inducing cell cycle arrest in the G2/M phases and loss of viability. These mechanisms are not selective for a specific DNMT, but rather involve all DNMTs acting on DNA.

Among the DNMTs, DNMT1 is normally referred to as the methylation maintenance enzyme, mainly ensuring the faithful transmission of the CpG methylation patterns from the parent to the daughter cells upon division. DNMT3A and 3B are involved in *de novo* methylation, particularly during development [19]. Nevertheless some reports suggest that DNMT3B could play a role in methylation maintenance as well [20, 21]. Still the respective role of each DNMT in promoting and maintaining the oncogenic transformation is largely unknown. Several articles have reported the use of RNA interference or knockout approaches to address the relative importance of each DNMT on DNA methylation, gene expression and cell proliferation, but essentially in solid tumors. For instance, the knockout of *DNMT1* or *DNMT3B* alone in the human colon cancer cell line HCT116 does not impact DNA methylation, while their concomitant invalidation induces profound hypomethylation leading to a minimal methylation footprint on DNA [22, 23]. In the same cell line, Robert *and coll*. found that DNMT1 depletion by siRNA induces CDKN2A demethylation [24], while another group using the same approach in the same cell line did not observe any demethylation of this gene [21]. DNMT3B was also shown to be required for the maintenance of DNA hypermethylation in cervical cancer cells [25]. In breast cancer cells, Chick *and coll*. demonstrated that the concomitant downregulation of DNMT1/3A/3B proteins lead to inhibition of cell growth and DNA hypomethylation associated with gene re-expression [26]. Interestingly, DAC leads to a different gene expression pattern compared to the siRNA-mediated depletion of all three DNMTs, suggesting potential additional targets for DAC-mediated phenotypes. Thus, despite a consistent number of studies, the importance of each DNMT for cancer maintenance still needs to be clarified. Noteworthy, it seems to depend on the cancer type, and, since all these data come from solid tumors, when FDA-approved demethylating agents are used in MDS and AML, we chose to explore the role of each DNMT in this pathology. To the best of our knowledge, concerning hematological cancers, the recent work from Peters *and coll*. is unique in relating the oncogenic potential of DNMT1 in a mouse model of MYC-induced T-cell lymphoma [27]. Here we addressed the role of each DNMT on the DNA hypermethylation and gene repression of TSG in the human AML KG1 model cell line, by using the RNA interference approach and compared it to the effect induced by DAC.

Our data show that (i) DNMT1 is the main methyltransferase involved in the maintenance of global and TSG promoter-specific (*TP73*, *CDH1* and *CDKN2B*) DNA hypermethylation in KG1 cells; (ii) DNA damage formation can be dissociated from DNA demethylation activity in cells exposed to DAC; (iii) KG1 cells express DNMT1 protein at a level largely superior to the one required to ensure DNA methylation maintenance; (iv) a threshold must be bypassed in order to trigger global DNA hypomethylation and specific DNA demethylation of tumor suppressor genes, and (v) *TP73* mRNA re-expression by DAC requires, in addition to demethylation of its promoter, another molecular event, potentially DNA damage.

## RESULTS

### SiRNA-mediated downregulation of each DNMT (DNMT1, 3A, and 3B) or concomitantly of the three together does not induce DNA demethylation, unlike the DNA demethylating agent DAC

First, the expression levels of DNMT1, 3A and 3B and their variant types were assessed in three blood cancer model cell lines, namely KG1, HL60 and Karpas299. All three *DNMTs* are expressed at the mRNA level (Supplementary Figure S1). However, only KG1 expresses relatively high levels of all DNMTs and especially of *DNMT3B*, compared to the other cell lines (Supplementary Figure S1 and Table S1). In particular, KG1 cells mainly express variant 6 of *DNMT3B* and several mRNA variants of *DNMT3A* (Supplementary Table S1). Because KG1 expresses all three *DNMTs* at high levels and it is known to possess several hypermethylated TSG promoters, we chose it as *in vitro* human leukemia model to address the respective role of each DNMT in the maintenance of DNA methylation homeostasis.

**Figure 1:**
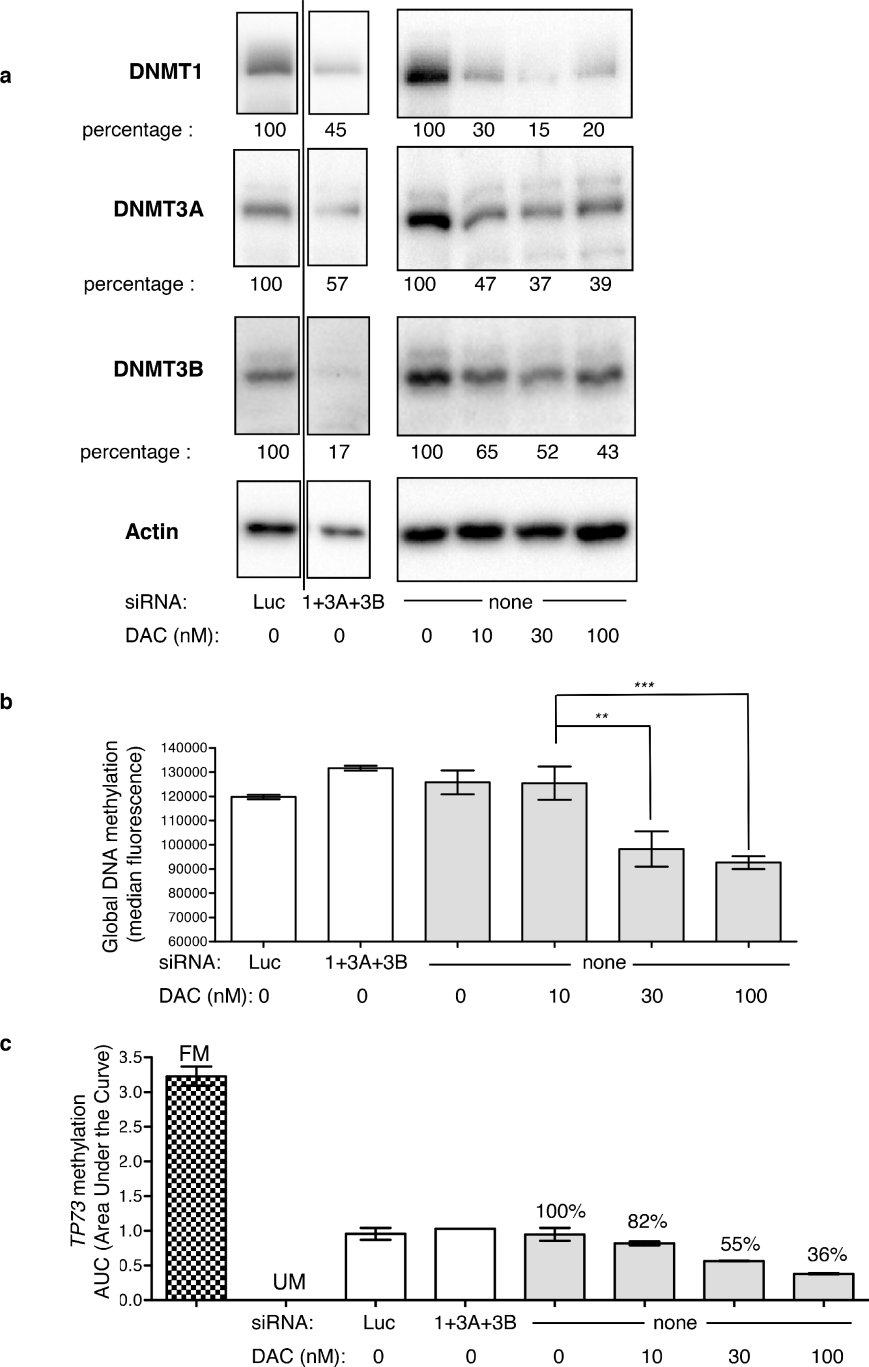
Impact of DNMT downregulation on global DNA methylation and TP73 promoter methylation **(a)** Western blot of DNMT1, 3A and 3B in KG1 cells treated with the combination of siRNAs directed against *DNMT1* (100 nM), *DNMT3A* (300 nM) and *DNMT3B* (300 nM), compared to siRNA directed against luciferase (Luc, at 700 nM)), and to a dose range of DAC (10, 30 and 100 nM). Actin protein was revealed on the same membranes and used to normalize for loading variations. The bands shown in the first quadrants (siRNA treated cells) have been cropped and come from the same gel (the full gels are accessible in Supplementary Figure S2). The relative percentages for each DNMT protein in response to the treatments are indicated (in %) and are the mean values of two independent experiments. The image shown is representative of one of these two. **(b)** The same cells were analyzed for total DNA methylation by flow cytometry (white bars cells treated with siRNA, gray bars cells treated with DAC). *P*-value: ** ≤ 0.01 and *** ≤ 0.001. **(c)** *TP73* promoter region methylation was assessed by MS-HRM analysis. The areas under the curves are reported for siRNA treated cells (white bars) and DAC-treated cells (gray bars). FM, fully methylated DNA control, UM, unmethylated DNA control.

**Figure 2:**
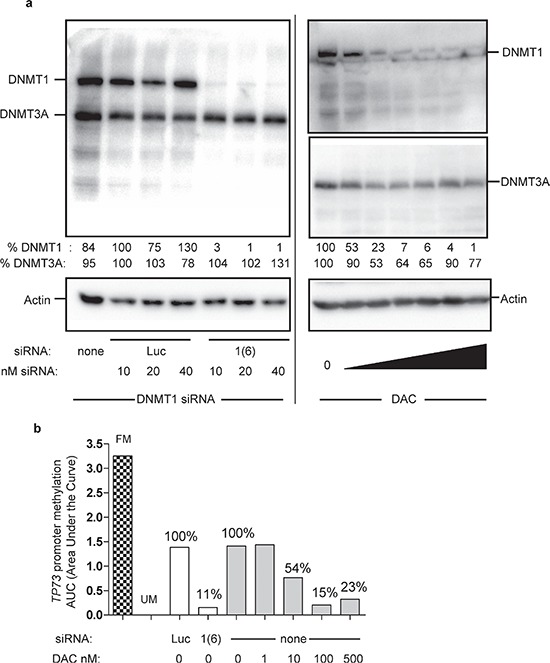
Impact of extensive DNMT1 depletion in HCT116 cells on *TP73* promoter methylation HCT116 human colon cancer cells have been transfected every two days for four days with the same DNMT1 siRNA (1(6)) used in KG1 cells, or exposed for 72 hours to a dose range of DAC (0, 0.001, 0.01, 0.03, 0.1, 1, 10 μM). **(a)** Two days after the first transfection, total proteins were extracted and subjected to Western blotting with DNMT1 and DNMT3A antibodies. Actin was used as loading control. The percentages of residual DNMT protein levels are indicated relative to the corresponding untreated controls normalized to 100% **(b)** Four days post-transfection DNA was extracted and subjected to MS-HRM analysis to evaluate the impact of DNMT1 depletion on *TP73* promoter methylation. In parallel with the siRNA treatment, HCT116 cells were exposed to a dose range of DAC for 72 hours (fresh DAC was added every day). FM, fully methylated DNA control, UM, unmethylated DNA control.

Second, each *DNMT* was downregulated by RNA interference (siRNA) and DAC was used as the reference DNA demethylating agent. Each designed siRNA depleted its corresponding DNMT - although to a different extent - without significantly affecting the expression of the other proteins (Supplementary Figure S2). When the three siRNAs were combined together, DNMT1/3A/3B were all depleted to similar levels, with mean residual percentage amounts of 45% (±17%), 57% (±16%), and 17% (±10%) for DNMT1, 3A and 3B, respectively (Figure 1a). Upon treatment with 100 nM DAC daily during 3 days, DNMT1, 3A, and 3B proteins were depleted down to 20% (±17%), 39% (±9%) and 43% (±12%), respectively. Noteworthy, the low dose of 10 nM DAC efficiently depleted each DNMT down to 30% (±13%), 47% (±19%) and 65% (±23%) for DNMT1, 3A and 3B respectively. The depletion induced by the siRNAs was slightly weaker compared to that induced by DAC for DNMT1 and DNMT3A but stronger for DNMT3B. Next we addressed the impact of these treatments on global DNA methylation (Figure 1b) and observed that DAC induced a significant DNA demethylation, while the downregulation by siRNA of either *DNMT1, DNMT3A* or *DNMT3B* individually or together did not affect significantly DNA methylation (Figure 1b, and data not shown for individual siRNA). Interestingly, the lowest dose used of DAC, 10 nM, hardly affected the level of global DNA methylation, although depleting already strongly the DNMTs. The impact of these DNMT depletion on the methylation of the promoters of three TSGs, *TP73*, *CDKN2B*, and *CDH1*, was investigated using Methyl-Specific High Resolution Melting (MS-HRM). As for global DNA methylation, DAC induced a dose-dependent demethylation of all three promoters, while siRNA-mediated depletion of the DNMTs did not show any significant effect (Figure 1c and Supplementary Figure S3 for *CDH1* and *CDKN2B*). Since three days of *DNMT* downregulation were not sufficient to observe an impact on DNA methylation, we double-transfected the siRNAs to increase the efficiency and duration of DNMT depletion. Only siRNAs targeting *DNMT1* and *3B* were used since they were more efficient than *DNMT3A* siRNA. Despite this longer downregulation period, promoter methylation of the three TSG was not affected, in contrast to DAC that led to a significant demethylation 72 h post-treatment and up to 4 days after discontinuing the DAC treatment (Supplementary Figure S4). Since it has been shown in the human colon cancer cell line HCT116 that downregulation of *DNMT1* by siRNA induced a demethylation of the promoter of *CDKN2A* [24], we tested in HCT116 the same *DNMT1* siRNA that we used in KG1. In this solid tumor cell line, a strong depletion of the DNMT1 protein was obtained (> 95%) similar to that obtained with 30 nM and higher concentrations of DAC (Figure 2a). This strong depletion induced a significant demethylation of the *TP73* promoter (Figure 2b) and of the *CDKN2A*, *IRF8* and *RASSF1A* promoters (data not shown), in agreement with the previous results. In addition, the strong depletion of the DNMT1 protein by the siRNA induced also a global demethylation at the LINE-1 repeated elements. Interestingly, lower doses of DNMT1 siRNA that induced a partial (40%) depletion of the protein had no global demethylating effect (Supplementary Figure S5). Consequently, we hypothesized that the partial siRNA-dependent DNMT downregulation (55% for DNMT1, 43% for DNMT3A and 83% for DNMT3B proteins) observed in KG1 may be the limiting factor for an efficient promoter demethylation. To explore this hypothesis and increase the depletion of DNMT, we combined the siRNA approach with 10 nM of DAC, a dose inducing depletion of all three DNMT, but without a marked impact on DNA methylation (Figure 1).

### Combining DNMT1 siRNA and DAC treatment increases DNMT1 depletion and induces global DNA demethylation

**Figure 3:**
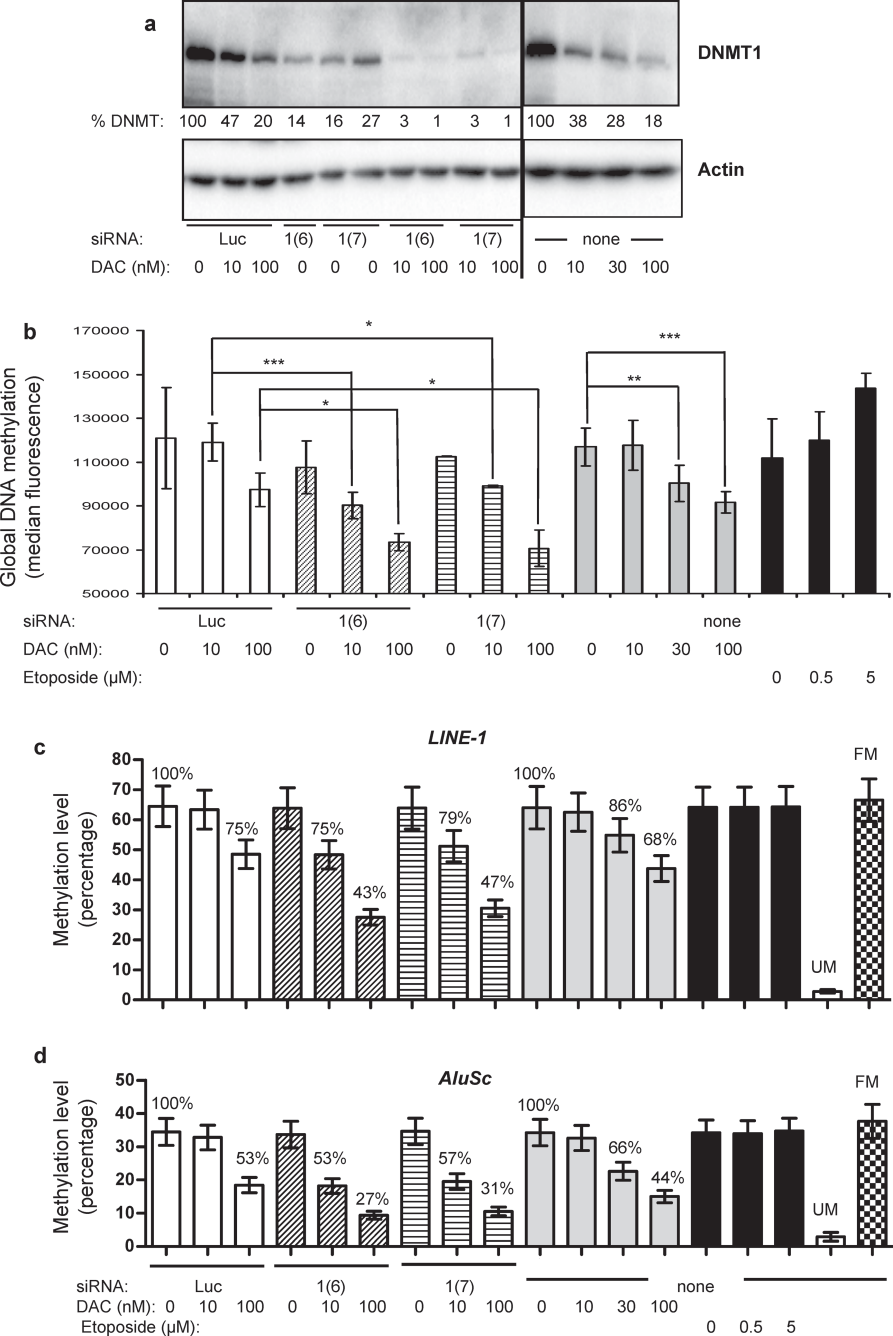
Effect of combining *DNMT1* siRNA and DAC on global DNA methylation and on the methylation of *LINE-1* and *AluSc* repeated sequences **(a)** DNMT1 Western blot performed on KG1 cells exposed to combination of siRNA (*Luc*, *DNMT1*(6) or *DNMT1*(7)) and two doses of DAC (10 and 100 nM) (left), compared to DAC-treated cells at 10, 30 or 100 nM (right). The full gel images are accessible in Supplementary Figure S6. The percentages of DNMT1 protein level (% DNMT1) normalized to 100% for their respective control (si*Luc* for electroporated cells and non treated for DAC treatment) are indicated. The image shown is representative of two independent experiments, except for the siRNA 1(7) that was tested in duplicates in this experiment **(b)** 72 h post-treatment, cells were collected and total DNA methylation was measured by flow cytometry for cells treated with siRNA *Luc* (100 nM) alone or combined with 10 or 100 nM DAC (white bars), siRNA *DNMT1*(6) (100 nM) alone or combined with 10 or 100 nM DAC (hatched bars); siRNA *DNMT1*(7) (100 nM) alone or combined with 10 or 100 nM DAC (striped bars); DAC alone at 10, 30 and 100 nM (gray bars) or etoposide at 0.5 or 5 μM (black bars). *P* value: * ≤ 0.05, ** ≤ 0.01 and *** ≤ 0.001. **(c–d)** KG1 cells treated for 72 hours with various combinations of DNMT1 siRNA (1(6) and 1(7)) and DAC were analyzed by bisulfite conversion followed by pyrosequencing for methylation changes in *LINE-1* (c) and *AluSc* (d). Cells were treated also with increasing concentration of etoposide. The mean methylation level of all CpG present in the sequenced DNA and the standard error are represented. The percentage of demethylation (normalized to 100% for the controls, *i.e*. *luciferase* siRNA and untreated cells) are also indicated. FM, fully methylated DNA control, and UM, unmethylated DNA control.

The combination of siRNAs targeting each DNMT with 10 nM DAC increased the level of DNMT1 depletion (> 95%), but not of DNMT3A and 3B depletion (Figure 3a and data not shown). Noteworthy, the control siRNA (*Luc*) had no modifying effect on the DNMT level, either used alone or combined with the increasing doses of DAC. We thus pursued our investigations with the *DNMT1* siRNA combined with DAC and used two different siRNAs, namely 1(6) and 1(7), targeting distant regions on the *DNMT1* mRNA sequence (see Materials and Methods). Both siRNAs alone induced similar depletion of the protein (Figure 3a), namely around 73–86%, and more than 95% when combined with 10 or 100 nM of DAC (Figure 3a). Then the impact of this DNMT1 depletion on global DNA methylation was measured. As shown in Figure 3b, combining *DNMT1* siRNA (1(6) or 1(7)) with 10 nM DAC induced a significant demethylation, similar to that induced by higher doses of DAC alone, 30 and 100 nM (Figure 3b). This was not observed with the siRNAs against *DNMT3A* and *3B* (data not shown). This synergy was increased in combination with 100 nM DAC. These results were confirmed by bisulfite conversion followed by pyrosequencing of the *LINE-1* and *AluSc* highly repeated sequences used as surrogate markers for global DNA methylation (Figure 3c and 3d). Thus an increase in the depletion of DNMT1 by combining the siRNA approach and low doses of DAC is able to demethylate DNA in KG1 cells. Since previous works suggested a potential role of DAC-induced DNA damage in the demethylation process [28], we explored the effect on DNA methylation of etoposide, a well-know DNA topoisomerase II poison leading to DNA breaks. Unlike DAC this compound was unable to induce any global DNA demethylation (Figure 3b). We then addressed whether this combination of treatments was able to demethylate specific TSG promoters and induce their re-expression.

### Combining *DNMT1* siRNA and DAC induces *TP73* promoter demethylation associated with gene re-expression

**Figure 4:**
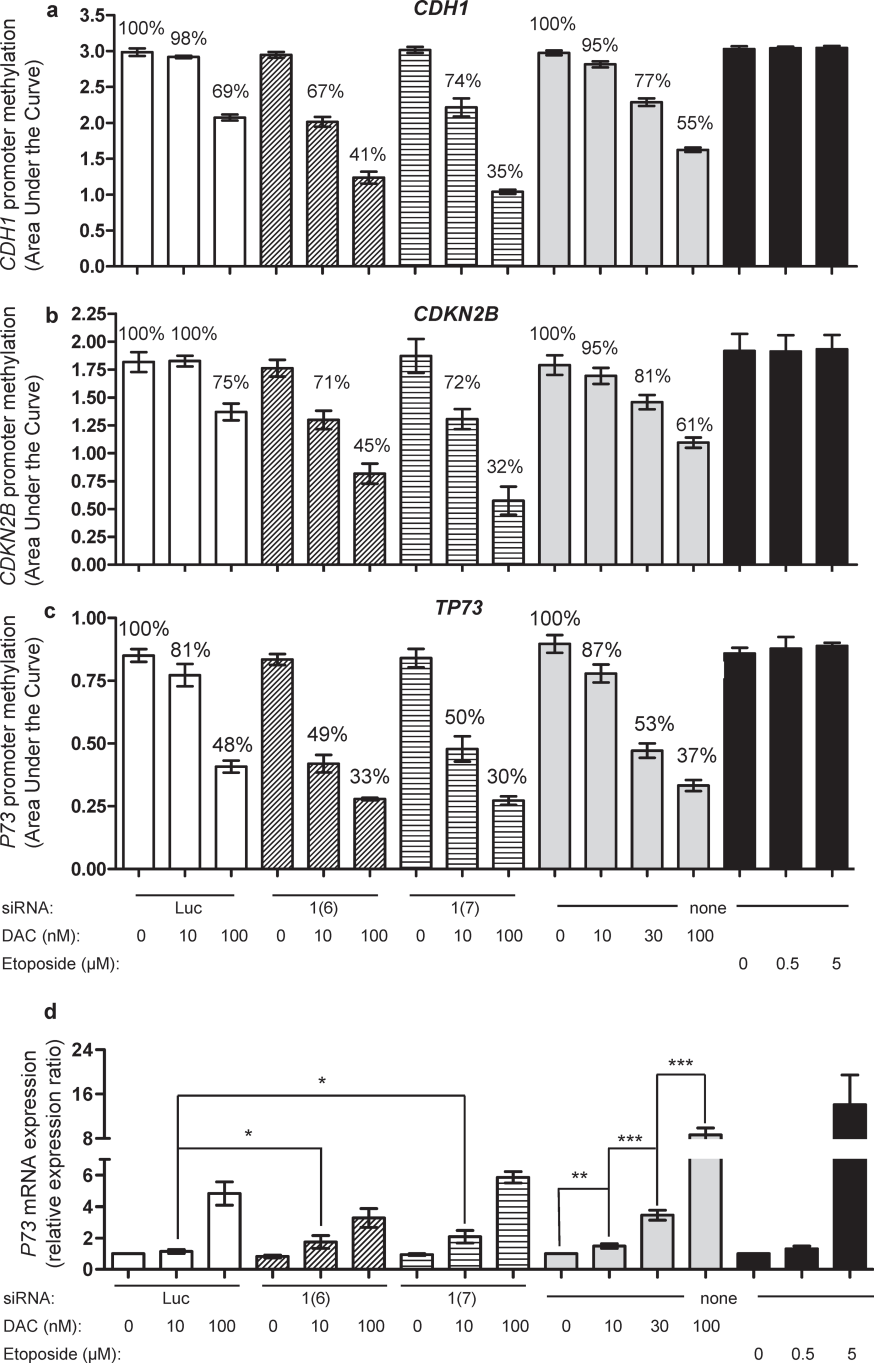
Impact of combining DNMT1 siRNA with DAC on promoter methylation of CDH1 and CDKN2B, and promoter methylation and expression of TP73 *CDH1* **(a)**, *CDKN2B* **(b)** and *TP73* **(c)** methylation was measured by MS-HRM 72 h after treating the cells with *DNMT* siRNA *Luc* (100 nM, white bars), siRNA *DNMT1*(6) at 100 nM (hatched bars), siRNA *DNMT1*(7) at 100 nM (stripped bars) and in combination with DAC (10 and 100 nM) (left), compared to DAC alone at 10, 30 and 100 nM (gray bars) or etoposide at 0.5 and 5 μM (black bars) (right). The mean percentage values of methylation, relative to the respective controls are indicated. **(d)** qRT-PCR analysis of *TP73* mRNA expression of the above treated cells. The expression ratio of the controls was normalized to 1, *i.e*., *luciferase* siRNA (for electroporated cells), water (the solvent of DAC) and DMSO (the solvent of etoposide), respectively. *P* value: * ≤ 0.05, ** ≤ 0.01 and *** ≤ 0.001.

As previously reported [29, 30], the promoter regions of three TSGs, namely *TP73*, *CDH1* and *CDKN2B*, are hypermethylated in KG1 cells (Figure 4 and Supplementary Figure S4). As expected, a dose range of DAC induced, after 72 h of daily treatment, a significant demethylation of all three genes, with 30 nM as the first significantly effective dose and achieving a stronger demethylation at 100 nM (Figure 4). In agreement with our observations on global DNA methylation, etoposide was unable to induce demethylation of the three promoters (Figure 4). Interestingly, the combined treatment of *DNMT1* siRNA with the demethylation-inactive dose of 10 nM DAC induced a strong and significant demethylation of all three TSGs, equivalent to higher doses of DAC alone (Figure 4). These results were obtained with both siRNAs against *DNMT1*. To further confirm these observations, promoter DNA methylation was analyzed by bisulfite conversion followed by pyrosequencing (Supplementary Figure S7), confirming the data obtained by MS-HRM. Noteworthy, in the case of *TP73*, pyrosequencing did not only confirm the demethylation activities seen by MS-HRM, but also assessed the impact of the combination on the methylation of the second promoter (P2) described for this gene (Supplementary Figure S8). Thus an extensive depletion of DNMT1 in KG1 cells, obtained by combining siRNA and DAC at low dose, induced global DNA demethylation and specific TSG promoter demethylation, among them *TP73*. This prompted us to study whether this demethylation affected *TP73* mRNA expression. DAC alone restored *TP73* expression in a dose-dependent manner 72 h after treatment as measured by qRT-PCR (Figure 4d). *DNMT1* siRNAs alone showed no effect, while when combined with 10 nM DAC, a modest but significant induction of *TP73* mRNA was measured (Figure 4d). Interestingly, even if the extent of DNA demethylation was equivalent, the extent of re-expression was lower (two-fold change) than with DAC alone at higher concentration (eight-fold change), implying that another mechanism induced by DAC is involved. Unexpectedly, when combined with 100 nM DAC, the siRNAs (*luc* and *DNMT1*) suppressed partially *TP73* gene re-expression induced by DAC alone at the same concentration (Figure 4d). Finally, etoposide induced *TP73* mRNA expression without having any impact on the methylation of the promoter, suggesting that DNA damage can induce *TP73* mRNA expression. Due to their very low expression level, *CDH1* and *CDKN2B* re-expression were not followed (data not shown).

### SiRNA-mediated DNMT1 depletion reverts the γH2AX phosphorylation and G2/M blockade induced by 100 nM DAC

**Figure 5:**
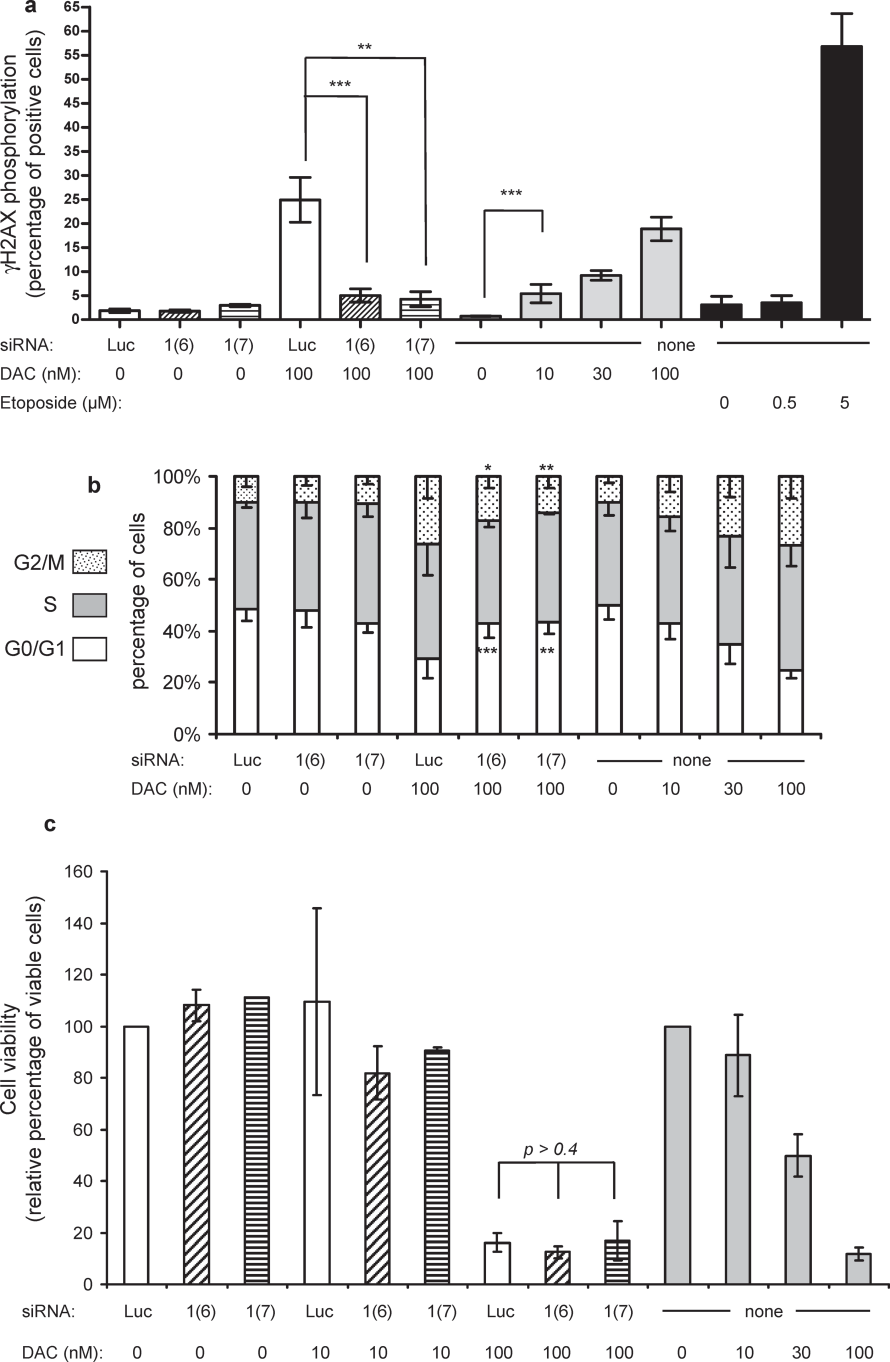
γH2AX phosphorylation labeling, cell cycle and cell viability analysis 72 h after treatment **(a)** The percentages of positive cells for γH2AX phosphorylation are reported for siRNA at 100 nM (si*Luc*, white bars, si*DNMT1*(6) hatched bars and si*DNMT1*(7) stripped bars) alone or in the presence of 100 nM DAC; for DAC alone at 10, 30 and 100 nM (gray bars) or for etoposide at 0.5 and 5 μM (black bars). *P*-value: ** ≤ 0.01 and *** ≤ 0.001. **(b)** The cell cycle repartition, indicated as percentages of cells in each phase (G0/G1 lower bars, S middle bars, G2/M upper bars), is shown for the various treatments applied to the cells. The stars on the histogram refer to the *p*-values calculated for both G0/G1 and G2/M compartments in KG1 cells treated with 100 nM DAC combined with either *DNMT1* siRNA1(6) and 1(7) versus the cells exposed to 100 nM DAC combined with the control siRNA (*Luc*). **(c)** After γH2AX phosphorylation measurement, cells from the same batch were seeded for another 4 days and cell viability assessed. The percentage of viable cells is indicated relative to their respective controls (si*Luc* and 0 nM DAC) set to 100% viable cells. White bars: 100 nM si*Luc*; hatched bars: 100 nM si*DNMT1*(6); stripped bars: 100 nM *DNMT1*(7); gray bars: DAC at 10, 30 and 100 nM.

Next we investigated the role of DNA damage induced by DAC at high doses, since etoposide, a DNA damaging agent, induced *TP73* re-expression and another mechanism than DNA demethylation seemed to be involved in *TP73* re-expression induced by DAC. The phosphorylation of γH2AX, a histone variant induced by DNA damage, was analyzed upon treatment of KG1 cells (Figure 5a). DAC alone induced a dose-dependent increase in the percentage of cells expressing phosphorylated γH2AX with 20% positive cells at 100 nM. Etoposide was used as positive control (57% positive cells at 5 μM), as it induces DNA double-stranded breaks through the formation of topoisomerase II-DNA covalent complexes, similar to the DNMT-DNA adducts formed upon exposure to DAC [13, 31, 32]. Both *DNMT1* siRNAs did not induce any significant phospho-γH2AX signal (Figure 5a). Interestingly, the combination of *DNMT1* siRNAs and 100 nM DAC strongly reduced the percentage of γH2AX phosphorylated positive cells (from 25% to less than 5%). Similarly, previously published data showed that knocking down topoisomerase II is associated with a reduced level of DNA damage induced by doxorubicin and etoposide [33, 34]. This is the result of a reduced formation of covalent adducts involving topoisomerase II, DNA, and the aforementioned topoisomerase II inhibitors. We then explored the consequences of the modulation of γH2AX phosphorylation on the cell cycle. In agreement with previous reports [16–18, 35], DAC induced a G2/M arrest in response to the activation of the G2/M checkpoint triggered by DNA damage (Figure 5b). Clearly, the *DNMT1* siRNAs reverted the G2/M blockade induced by 100 nM DAC (Figure 5b, and Supplementary Figure S9 for a focus on G0/G1 and G2/M phases). Finally, the impact on KG1 cell growth was explored. Since DAC is known to induce a delayed effect on cell proliferation [36], the cell viability was measured seven days after the first treatment (Figure 5c). As expected, DAC induced a dose-dependent loss of cell viability of 50% and 90% at 30 nM and 100 nM, respectively. Although clearly reversing γH2AX phosphorylation and G2/M blockade, siRNA-mediated downregulation of DNMT1 did not protect the cells from the loss of viability measured seven days after exposure to repeated DAC treatments (Figure 5c).

### A genome wide analysis of DNA methylation changes at 450 000 CpGs induced by the combination of *DNMT1* siRNA and DAC

**Figure 6:**
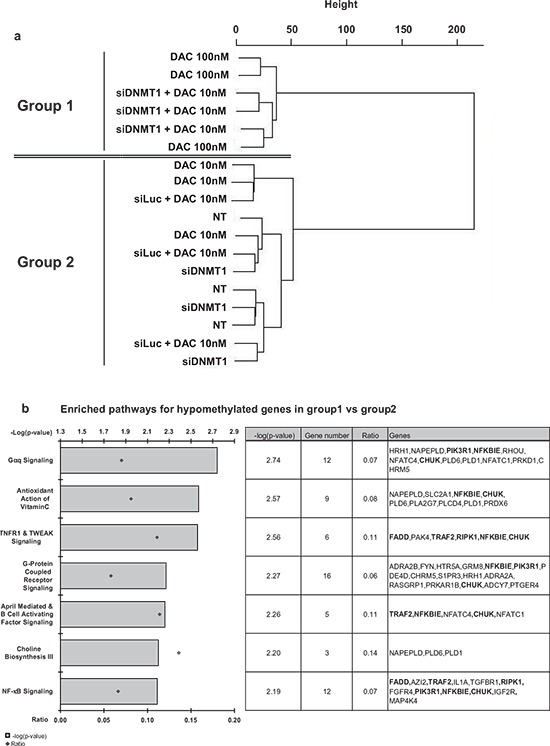
Genome-wide methylation analysis The genomic DNA extracted from treated KG1 cells was analyzed on an Illumina 450K CpG microarray. **(a)** The clustering analysis shows two groups: group 1 corresponding to cells treated by 100 nM DAC or the combination of siDNMT1(6) and 10 nM DAC; and group 2, corresponding to non-treated cells (NT), cells treated with 10 nM DAC, the combination of siLuc and 10 nM DAC or siDNMT1 alone. Biological triplicates have been performed for each condition. **(b)** IPA^®^ pathways analysis of hypomethylated genes enriched in group 1 *vs*. group 2. Bar plots, on the left, show the canonical pathways the most significantly represented in group 1 (threshold *p*-value < 0.01) and the ratio values corresponding to the proportion of deregulated genes among all genes found in each pathway. The table on the right gives a detailed view of the genes hypomethylated in each pathway. The genes involved in the NF-kB pathway are in bold.

Next, the effect of the combination of the siRNA against *DNMT1* with a low dose of DAC was explored at the genome-wide level using the Illumina 450K CpG BeadChips. Unsupervised clustering analysis confirmed that cells treated with the high concentration of 100 nM DAC sustain similar methylation changes than those treated with the low dose of DAC (10 nM) combined with the *DNMT1* siRNA (group 1 in Figure 6a). Non-treated cells, cells treated with low concentration of DAC and cells transfected with DNMT1 siRNA alone clustered together in group 2 (Figure 6a). Since it is known that DNA methylation of promoters has a direct role in genes transcription, we focused our analysis on the genes for which the modulated CpG are located within, or in a close vicinity, of their corresponding transcription start site (TSS). We identified 689 hypomethylated genes in group 1, including *CDKN2B*, in agreement with our MS-HRM and pyrosequencing data. Most surprisingly, we observed also that 1 254 genes were hypermethylated in this same group 1 (Supplementary Table S2 and S3 for the lists of hypo- and hypermethylated genes, respectively). Next, we analyzed the pathways that were mostly affected by hypomethylation changes in group 1, using the Ingenuity Analysis Pathway^®^ (IPA^®^) tool. The most significantly affected pathways (*i.e*., with the lower *p*-values, -log(*p*-value) > 2) concerned major cell signaling pathways that share several common factors with NF-kB network (Figure 6b, genes in bold in the table). This observation suggested that main apoptosis/proliferation functions are affected by hypomethylation following DNMT1 strong depletion (100 nM DAC or 10 nM DAC combined with siDNMT1).

## DISCUSSION

**Figure 7:**
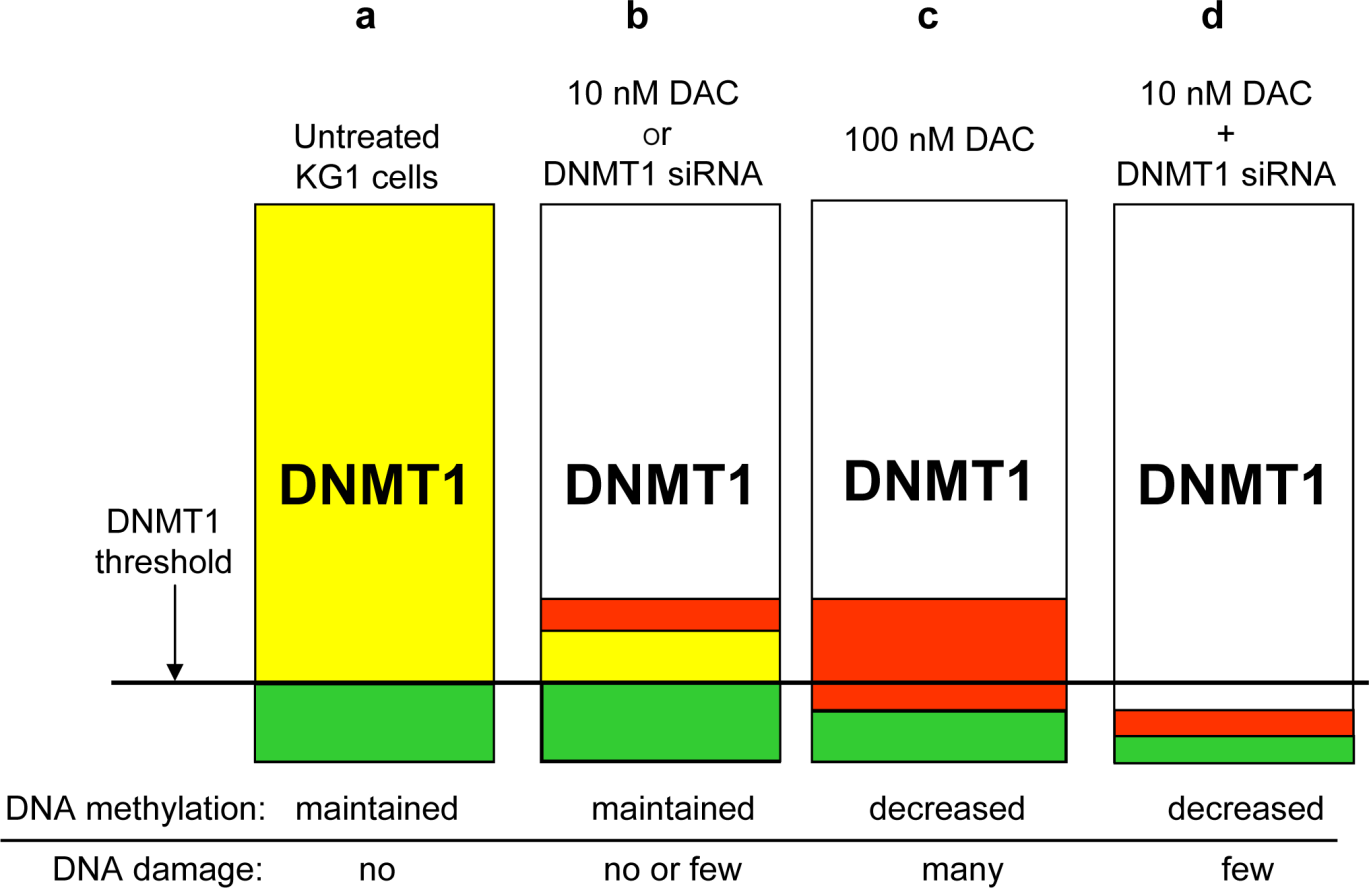
Schematic representation of the DNMT1 threshold hypothesis for DNA methylation maintenance **(a)** In KG1 cells the physiological level of DNMT1 is largely above the minimum amount to ensure proper DNA methylation propagation, defining a reservoir of protein (yellow). **(b)** The partial loss of DNMT1, either by a low dose of DAC (10 nM) or a siRNA specific to *DNMT1*, reduces the DNMT1 content, but the remaining free pool (green + yellow) is sufficient to maintain the CpG methylation, even though a small proportion of detected DNMT1 can be trapped by the DAC incorporated into DNA (red), leading to few DNA breaks, which is not the case for the siRNA. **(c)** A higher dose of DAC (100 nM) strongly lowers the free DNMT1 pool below the critical threshold, triggering global and specific DNA demethylation. At the same time, DNMT1-DAC adducts formed on DNA translate into DNA breaks visualized by γH2AX phosphorylation. **(d)** Depletion of the DNMT1 pool, below the threshold, can also be achieved by combining a low dose of DAC with a siRNA directed against *DNMT1*. Similar to a high dose of DAC (*i.e*. 100 nM) this combination induces a global and gene-specific DNA demethylation, but little DNA damage, since less DAC and also less DNMT1, downregulated by the siRNA, are present to form the DNMT-DNA adducts and consequent DNA damage. Yellow box: Reservoir of DNMT1 not required to maintain DNA methylation Green box: Pool of DNMT1 required to ensure DNA methylation maintenance Red box: Pool of DNMT1 that can be trapped on DNA by DAC and be converted into DNA damage DNMT1 threshold = defines the minimal pool of DNMT1 required for DNA methylation maintenance

Our data show that all three DNMT1/3A/3B are expressed in KG1 cells, with DNMT1 and 3B being predominant. This is in agreement with previous data on CD34^+^ leukemia cell lines, such as KG1, showing high expression of DNMT3B [37]. Surprisingly, partial DNMT downregulation by siRNA in KG1 cells (60 to 90%) did not induce any global or specific DNA demethylation, in contrast to what has been observed in HCT116 cells, where we could achieve a DNMT1 depletion > 95%. To explain these apparent discrepancies, we suggest that KG1 cells express high levels of the three DNMT1/3A/3B, and particularly of DNMT1, which exceeds the minimum amount required to maintain CpG methylation (Figure 7). This minimal amount defines the threshold, below which DNA demethylation is observed (Figure 7a). First, while in 10 nM DAC co-treated cells the DNMT1 that is not depleted can be trapped on the DNA by the incorporated DAC (Figure 7b), in the siRNA treated cells all residual DNMT1 is mobilized to ensure DNA methylation (Figure 7b). This explains why the siDNMT1 are unable to demethylate DNA in KG1 cells. In agreement with this hypothesis Patel *and coll*. showed that in DAC treated cells the formation of cytosine adducts are more critical for DNA demethylation than the residual level of DNMT1 [38]. Second, in the case of *DNMT1* siRNA combined with a low dose of DAC, the free available pool of protein drops below the minimally required amount and induces DNA demethylaton, but with reduced DNA damage (Figure 7d) compared with 100 nM DAC (Figure 7c). This combined treatment allows then to reveal the passive demethylation component of DAC, in contrast to the active demethylation involving the formation of DNA damage. Nonetheless, we cannot rule out the possibility that the low dose of DAC used (10 nM) in combination with *DNMT1* siRNA could participate in a more active manner than just lowering the level of DNMT1 available for DNA methylation. In conclusion, this study reveals an essential role of DNMT1 in maintaining DNA hypermethylation in KG1 cells, in contrast to DNMT3B, which is abundant in KG1. Regarding DNMT3A, its expression is already low in KG1 cells. It is also possible that DNMT3A and/or DNMT3B play other roles in addition to DNA methylation management, since Hagemann *and coll*. recently reported that DNMT3B depletion in human colon cancer cells induced cell growth arrest and apoptosis without changes in their DNA methylation pattern [39].

Concerning the role of DNA damage in DNA demethylation, it has been suggested that formation of the suicide complex between incorporated DAC and DNMT participates to DNA demethylation through a DNA repair mechanism, a process referred to active demethylation [28]. Here we clearly separated DNA damage formation and DNA demethylation by combining *DNMT1* siRNA and low doses of DAC, since this combination induced DNA demethylation with lower DNA damage compared to DAC alone at 30 nM or 100 nM. Interestingly, two recent studies showed that depleting either DNMT1 or DNMT3B in cancer cells protects them from gene misregulation and cell cycle arrest induced by a DAC treatment, hence supporting our present findings [14, 15]. Accordingly, the suppressive effect of *DNMT1* siRNA on DAC-mediated *TP73* mRNA induction that we observed could well be explained by a significant reduction of DNA damage, through lowering the DNMT1-DNA adduct formation that would participate, at least in part, in the gene re-expression.

Another main conclusion is that the antiproliferative effect of DAC does not seem to be associated with its ability to induce DNA breaks. Indeed, the *DNMT1* siRNAs combined with low doses of DAC, which induced the same DNA demethylation as high DAC doses but with lower DNA damage, had the same cell viability inhibition at 7 days as DAC at high doses. This result suggests that the induced DNA demethylation plays an important role in affecting cell growth, possibly by the re-expression of TSGs, ultimately leading to cell cycle arrest and cell death. We thus succeeded in turning the initial drawback of a partial DNMT1 depletion by siRNA in KG1 cells into an advantage by combining this approach with low dose of DAC, giving insights into the mechanism of action of DAC and one of its main targets, DNMT1.

To further compare the effect of the downregulation of DNMT1 by DAC or by the combination of siRNA and non-demethylating doses of DAC, we performed a microarray analysis to explore the methylation changes in the genome. Again the two treatments that strongly depleted DNMT1 clustered together compared to the controls, 10 nM DAC or *DNMT1* siRNA alone. Interestingly, in parallel to a general hypomethylating activity, we observed promoter hypermethylation of certain genes. Nevertheless, we cannot exclude that different cell populations are present in the bulk and behave differently. The analysis of the pathways that are hypomethylated in the treated KG1 cells compared to the non-treated converges on genes related to several cell signaling pathways as targets of the combination of DAC and DNMT1 siRNA (Figure 6). The NF-kB network (*i.e*. FADD, TRAF2, RIPK1, PIK3R1, NFKBIE and CHUK, in bold in Figure 6b) is clearly affected and may be involved in the antiproliferative activity of this combination.

Finally, two recent articles report the successful TSG induction in cancer cells by combining DAC with the inhibition of two other epigenetic modifiers, HDAC and LSD1 [40, 41]. These data combined with our present results support the rationale to test, in leukemia models, the combined treatment of DNMT1 selective inhibitors with already approved “epidrugs”, such as HDAC inhibitors, or preclinically investigated molecules such as LSD1 inhibitors. Importantly, our data suggest that combining low doses of DAC with a DNMT1 selective inhibitor could achieve an efficient TSGs hypomethylation associated with a reduced impact on DNA damage and potentially reduce the side effects observed with high doses of DAC.

## METHODS

### Cell lines and reagents

KG1, HL60, Karpas299 and HCT116 cell lines were obtained from the ATCC repository (Manassas, VA, USA), and cultivated in RPMI1640 medium (BE12–115F, Lonza, Basel, Switzerland) complemented with 10% fetal calf serum (Gibco by Life Technologies, Cergy Pontoise, France), and under 5% CO_2_. 5-Aza-2′-deoxycytidine (DAC) and etoposide were obtained from Sigma Aldrich (Saint-Quentin Fallavier, France), and prepared as 10^−2^M aliquots in H_2_O and DMSO respectively, stored at −20°C and used as dilutions freshly prepared in culture medium. The siRNAs used to target *DNMTs* and *luciferase* were purchased from Qiagen (Courtaboeuf, France) and Fisher Scientific (Illkirch, France) respectively and stored at −80°C before use.

### siRNA delivery

In the case of KG1, the 4D-Nucleofector™ System (Lonza) was used to vector one or several siRNAs. In a typical experiment, 8×10^6^ actively growing KG1 cells were transfected in 100 μL SF solution with 100 nM (*DNMT1*) or 300 nM (*DNMT3A* or *3B*) siRNA according to the manufacturer's instructions. Corresponding concentrations of *luciferase* siRNA were used as negative controls. Immediately after electroporation cells were collected in 5 mL fresh medium and incubated for the indicated time periods. The GFP-expressing vector pmaxGFP from Lonza was used to assess the transfection efficiency by flow cytometry 24 h post-treatment. Usually 70 to 80% cells expressed GFP. For HCT116, Lipofectamine^®^ RNAiMAX Transfection Reagent from Invitrogen (by Life Technologies) was used to deliver DNMT1 or *luciferase* siRNA to the cells. Briefly, a premix of 10 μL siRNA (diluted in water to ensure final concentrations of 10, 20 and 40 nM) with 485 μL OptiMEM (Invitrogen by Life Technologies) and 5 μL transfection reagent was incubated for 25 min. 250 μL of this suspension was mixed with 1.5 × 10^5^ cells and seeded in a 6 well plate. After the indicated period of time the cells were trypsinized for further analysis. Sequences of siRNAs used to target DNMTs:

**Table:** 

TARGET	sequence	Qiagen reference	Position (RefSeq) on mRNA variants (V)
*DNMT1(6)*	F: CAAUGAGACUGACAUCAAATT R: UUUGAUGUCAGUCCUCAUUGGG	SI02663409	V1: 3253–3273 V2: 3205–3225
*DNMT1(7)* *DNMT1(7)*	F: GGAAGUGAAUGGACGUCUATT R: UAGACGUCCAUUCACUUCCCG	SI02663416	V1+V2: 481–501
*DNMT3A*	F: CAGUGGUGUGUGUUGAGAATT R: UUCUCAACACACACCACUGAG	SI02665278	V1: 1346–1364 V2: 559–577 V3: 1275–1293 V4: Non targeted
*DNMT3B*	F: GCUCUUACCUUACCAUCGATT R: UCGAUGGUAAGGUAAGAGCTG	SI03068240	V1: 807–827 V2: 807–827 V3: 807–827 V6: 786–806 V7: 681–701 V8: 579–599

As a control, we used the *luciferase* siRNA #P0020990120 from Fisher Scientific herein abbreviated as *Luc* or *Luc* siRNA. Similarly, and for clarity reason, the other siRNAs directed at *DNMT1*, *3A* and *3B*, are referred in the figures legends as 1 or 1(6), 1(7), 3A and 3B respectively.

### Quantitative Real-time PCR (qRT-PCR)

The cDNA, obtained from 1 μg total RNA by using the SuperScript^TM^ II Reverse Transcriptase following the manufacturers instructions (Life Technologies) coupled to the random primer from Promega (Charbonnières- les-Bains, France), were used at 1/25 dilution to perform real-time PCR reactions with a SYBR^®^ Green PCR Master Mix kit from Applied Biosystems (by Life Technologies), using 400 nM of each forward (F) and reverse (R) primer obtained from Eurogentec (Angers, France). We used a CFX384 Touch™ Real-Time PCR Detection System from Biorad (Marnes-la-Coquette, France) to run the following PCR program: 95°C 10 min followed by 40 cycles of 15 sec at 95°C, 1 min at 65°C for elongation, ended with a fusion cycle to determine the Tm of each amplification product. The PCR data were analyzed with the CFX Manager v3.0 software from Biorad to generate the Ct values and relative expression ratios. The following quality controls were applied: amplification of a single product, no amplification in the NRT (No reverse transcription) condition, efficiency close to 100%, and R^2^ above 0.98. The 2^−ΔΔCt^ method was used to generate the gene expression ratios, and the mean Ct values from three reference genes, *i.e*. *PPIA*, *TBP*, and *YWHAZ* were used to normalize for cDNA input variations [42].

**Table:** Sequences of real-time PCR primers

TARGET	sequences	Position (RefSeq) on mRNA and amplified variants (V)
*TP73*	F: GGACGTACTCCCCGCTCTTGA R: TCCGCTTTCTTGTAAACAGGCATG	All 13 variants are theoretically amplified^$^
*PPIA*	F: GAGCACTGGAGAGAAAGGATTTGGTT R: CGTGTGAAGTCA-CCACCCTGACA	200–289
*TBP*	F: TTGACCTAAAGACCATTGCACTTCGT R: TTACCG-CAGCAAACCGCTTG	610–678 (V2)
*YWHAZ*	F: CCCTCAAACCTTGCTTCTAGGAGA R: TCATATCGCTCAGCCTGCTCG	168–214 (V4)

$ For clarity reason the nucleotide positions on each TP73 amplicon are not indicated.

### Western blots

SDS-PAGE was performed to measure the relative expression of DNMT1, 3A and 3B according to the procedure described by UK Leammli [43]. Briefly, 48 h after siRNA transfection or DAC exposure, 20 μg total protein were loaded onto 8% polyacrylamide gel, separated through electrophoresis, transferred onto PVDF membrane, and incubated with the following primary antibodies, at the indicated dilutions: mouse anti- pan-actin 1:40 000 (MAB1501, Millipore, USA), rabbit anti-DNMT1 1:10 000 (NB100–264, Novus Biologicals, Littleton, CO, USA), rabbit anti-DNMT3A 1/500 (3598, Active Motif, Carlsbad, CA, USA), goat anti-DNMT3B 1:100 (N-19, sc-10235, Santa Cruz Biotechnology, Dallas, TX, USA). These antibodies were revealed with secondary HRP-coupled antibodies from Sigma Aldrich. All the incubation steps were performed in the following buffer: 200 mM NaCl, 20 mM TrisHCl pH7.6, 0.1% Tween 20. The Immobilon Western Chemiluminescent HRP Substrate from Merck Millipore (Darmstadt, Germany) was used to reveal the protein luminescent signals acquired on a ChemiDoc™ MP gel imaging system from Biorad and subsequently quantified with the Image Lab v4.0.1 software from Biorad. The signals from actin were used to normalize the protein loading variations. The relative expression values for each DNMT are represented by percentages obtained by the following formula: DNMT signal intensity value divided by the corresponding intensity for the actin giving a ratio normalized to 100% for each control sample.

### Total 5-methylcytosine labelling and FACS analysis

The analysis of the total 5-methylcytosine (5mC) content in cells was performed according to the method described in Desjobert *et al*. [44]. Briefly, the cells were fixed with 4% paraformaldehyde in PBS, and then permeabilized in 0.5% Triton X-100 in PBS. After treatment with HCl 2N (30 min, 37°C) and neutralization with Tris HCl 100 mM pH 8.8, the cells were incubated in a blocking solution (1% BSA, 0.05% Tween 20 in PBS). The 5mC labelling was performed with a primary monoclonal antibody against 5mC (clone 33D3, AbD Serotec by Biorad) and an Alexa-Fluor^®^ 647-conjugated goat anti-mouse Ig secondary antibody (Invitrogen by Life Technologies). Cells were finally labelled in 5 μg/mL propidium iodide (PI) in PBS. All steps were performed at room temperature except when indicated.

The 5mC labelling was analyzed on a LSRII cytometer (BD Biosciences, Le Pont de Claix, France) using the BD FACSDiva software. After gating the cells according to their propidium iodide content in order to exclude debris, cell-doublets, aggregates or apoptotic cells, the means of fluorescence intensities (mfi) were recorded on at least 5000 cells. The 5mC mfis were reported to the mfis from control samples as indicated in the legends.

### DNA extraction and bisulfite treatment

DNA was isolated from cultured KG1 cells using the DNeasy Blood and Tissue Kit on a QIACUBE automation system according to the manufacturer's specifications (Qiagen). DNA bisulfite conversion was performed on 1 μg of DNA using the Epitect Plus DNA Bisulfite Kit according to the manufacturer's specifications (Qiagen). Cycling conditions on the PCR apparatus were defined by 3 cycles of denaturation/incubation with times/temperatures set as follows: 5 min at 95°C to denature the DNA followed by a 60°C incubation for 25, 85 and 175 min for each cycle. The clean-up step of bisulfite-converted DNA was carried out on a QIACUBE automation system according to the manufacturer's specifications (Qiagen).

### Methyl specific high resolution melting experiments (MS-HRM)

Primers, forward (F) and reverse (R), were designed using bisulfite-converted sequences of the promoters from the studied genes:

**Table:** 

Target	Primer sequences	Position Refseq (NCBI)
*CDH1*	F-GGAATTGTAAAGTATTTGTGAGTTTG R-ACTCCAAAAACCCATAACTAACC	chr16: 68771204-6877133 (F/R)
*CDKN2B*	F-CGTTTTTAGTTGGGTTAAGGGGT R-GTCCTAACATCTTTAAACAAACTTCCC	chr9: 22009329-22009464 (F/R)
*TP73*	F-GTTATATTTTTTGTTTTTTGGATTTTAAG R-TTTCCTAACACCCGAATCTCTCCT	chr1: 3568567-3568661 (F/R)

The localization of the primers is reported in Supplementary Figures S8, S10 and S11.

The PCR reactions were performed as duplicates in a final volume of 15 μL on 70 ng of bisulfite converted DNA as follows: 2 min at 95°C to activate the “hot start” enzyme and 45 cycles at 95°C for 10 sec and 58°C for 30 sec; followed by a high resolution fusion curve from 65°C to 90°C (0.2°C step climb every 10sec) to determine the specific Tm of each amplicon. Hypomethylation will translate into a lower Tm due to the transformation of unmethylated cytosines into uracils and thymidines after PCR. The MS-HRM PCR efficiency was higher than 90%. Data analyses were performed using the Precision Melt Analysis software (Bio-Rad). The melting curve of unmethylated control DNA was subtracted from the melting curve of each sample for data analysis and representation. This transformation allows us to obtain an Area-Under-Curve (AUC) plot correlated to the methylation rate of each studied promoter. Fully methylated (FM) and fully unmethylated (FU) control DNA were obtained from Qiagen.

### Pyrosequencing experiments

The preparative PCR with a biotinylated primer was performed on 20 ng of bisulfite-converted DNA with Pyromark PCR Master Mix (Qiagen). Reactions were carried out in a final volume of 15 μL as follows: 15 min at 95°C to activate the “hot start” enzyme and 45 cycles at 95°C for 30 sec, 56°C for 30 sec and 72°C for 30 sec; followed by a final extension at 72°C for 10 min. After denaturation of PCR products and binding of biotinylated-strands on sepharose coated streptavidine beads, pyrosequencing was performed on a Pyromark Q24 according to the manufacturer's specifications (Qiagen). Amplification and sequencing primers have been designed and manufactured by Qiagen under the following referenced assays, except for *LINE-1* and *AluSc* primers that were manually designed.

**Table:** Primers for Pyrosequencing

Target	Primer sequences	Position Refseq (NCBI)
*CDH1*	Hs_CDH1_02_PMPyroMarkCPGassay PM00171948	chr16: 68772100-68772129
*CDKN2B*	Hs_CDKN2B_01_PMPyroMarkCPGassay PM00039893	chr9: 22008945-22008981
*LINE-1*	sense 5ʹ-GGGTTTATTTTATTAGGGAGTGTTAGAT-3ʹ reverse-biotin 5ʹ-AAAAAAACTCCCTAACCCCTTAC-3ʹ sequencing 5ʹ-AGTGGGAGTAGGTTAGT-3ʹ	Repeated sequences
*AluSc*	sense 5ʹ-AGAGATAGAGATTATTTTGGTTAATATGG-3ʹ reverse-biotin 5ʹ-AATTCAAACCATTCTCCTACCTCAAC-3ʹ sequencing 5ʹ-ATTAAAAATATAAAAATTAGTTGG-3ʹ	Repeated sequences
*TP73*	Hs_TP73_02_PMPyroMarkCPGassay PM00084868	chr1: 3607097-3607129

Localization of the primers is reported in Supplementary Figures S8, S10 and S11.

### γH2AX phosphorylation and cell cycle analysis measurements

Seventy-two hours after treatment, 5 × 10^5^ cells were collected, fixed and permeabilized with DNA Prep LPR (6607055, Beckman Coulter), incubated with an Alexa Fluor 647-conjugated anti-γH2AX phosphorylated (Ser139) antibody from Biolegend (613408, London, United Kingdom) at a 1:150 dilution in PBS 1% BSA for 45 min. Cells were then washed with PBS 1% BSA, and incubated for 60 min with DNA Prep Stain solution (Beckman Coulter) to stain the DNA. Cells were then analyzed with a Becton Dickinson LSRII flow cytometer. ModFit LT™ 3.0 software was used to analyze the repartition of the cells along cell cycle phases*, i.e*., G0/G1, S and G2/M. For the γH2AX phosphorylation analysis, cells incubated with vehicle, or electroporated with the control siRNA, were used to define a fluorescent threshold for cells expressing the phosphorylated form of γH2AX.

### Cell survival

Briefly, 2×10^5^ KG1 cells, previously treated for 72 h with either various doses of DAC, *DNMT1* siRNAs or both, were seeded in 6 well plates. 96 h later cell viability was assessed via the Trypan Blue (Sigma Aldrich) exclusion method using a Cellometer^TM^ from Ozyme (St Quentin Yvelines, France).

### Genome-wide methylation profiling using CpG microarrays

DNA quantitation was measured using the Quant-iT^TM^dsDNA Broad-Range Assay Kit (Life Technologies) according to the manufacturer's instruction. 1 μg of DNA was bisulfite-converted using the EpiTect Bisulfite Conversion Kit (Qiagen). The Infinium^®^ 450K Methylation BeadChip (Illumina Inc., San Diego, CA, USA) was used for genome-wide methylation analysis. The experimental protocol was followed according to the manufacturer's instructions using 200 ng DNA of each bisulfite-converted sample. Bead Chips were scanned using the Illumina^®^ iScan system. The methylation level of each CpG site was calculated as the methylation beta value using the intensities between methylated and unmethylated probes (β-value = Methylated probe intensity (M) / (Unmethylated probe intensity (U) + Methylated probe intensity (M) + 100) as defined by Illumina. Data were extracted using the Genome Studio software version 2011.1, Methylation module version 1.9.0 (Illumina Inc.) without any normalization steps. As quality control, Infinium I/II shift correction and data normalization was performed using a refined version of an in-house developed pipeline based on subset quantile normalization [45]. Unsupervised hierarchical clustering was performed using β-values for distance calculation and the “Ward” agglomerative hierarchical clustering method as distance measure minimizing the total within-cluster variance using squared Euclidean distances. The differentially methylated genes between Group1 and Group2 were analysed by the Ingenuity Pathways Analysis^®^ tool according to the number of deregulated CpG sites in the genes TSS. The most enriched canonical pathways were considered significant with a threshold *p*-value < 0.01. The value of ratio is calculated by dividing the number of deregulated genes in a specific pathway by the total number of genes that constitute this pathway. All the data generated in this genome-wide analysis come from three biological independent experiments for each control and treatment analyzed.

### Statistical analysis

A minimum of two biologically independent experiments were performed to generate mean values +/– standard deviation (SD). In the case of global methylation measurement through flow cytometry, cell cycle analysis, and γH2AX phosphorylation, a minimum of 4 replicates were compiled due to the small variations between the various treatments. For the *p*-value calculations, and since more than two conditions had to be compared, one way ANOVA associated with the contrast method was used (SAS R9.3). One star * refers to a *p*-value ≤ 0.05, ** ≤ 0.01 and *** ≤ 0.001. In the case of MS-HRM, the *p*-values were obtained from Tukey's post-hoc comparisons.

## SUPPLEMENTARY FIGURES AND TABLES






