# Supplementary material for: Consequences of combining siRNA-mediated DNA methyltransferase 1 depletion with 5-aza-2′-deoxycytidine in human leukemic KG1 cells
Source: Oncotarget. 2015 Mar 20;6(17):15265–82. doi: 10.18632/oncotarget.3317 (PMC4558150; doi:10.18632/oncotarget.3317)
Supplement: Supplementary file 1 [file oncotarget-06-15265-s001.pdf]

SUPPLEMENTARY FIGURES AND TABLE

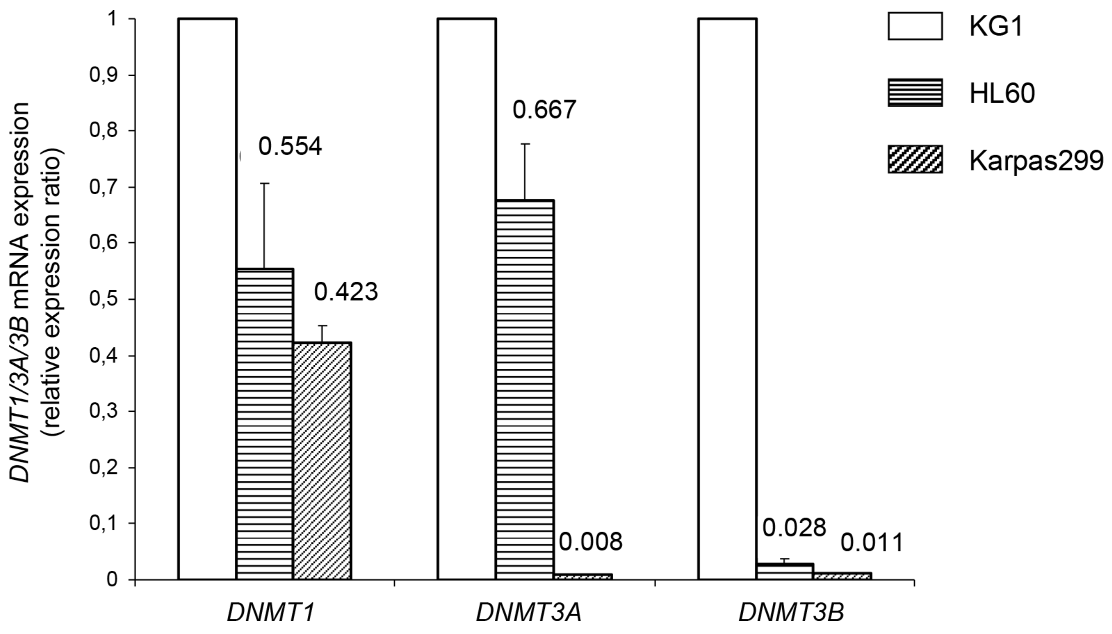

Supplementary Figure S1: Relative expression ratio of the three *DNMT1/3A/3B* mRNA in the three leukemia cell lines KG1, HL60 and Karpass299 cells. RT-qPCR reactions were performed on RNA extracted from growing KG1, HL60 and Karpas299 cells and the relative ratios of mRNA expression were determined as described in the Materials and Methods Section. The primers were chosen to amplify all or most of the variants for each *DNMT* and correspond to those that gave the lowest Ct values as reported in Supplementary Table S1. The expression ratios for HL60 and Karpas299 were calculated relative to KG1 set to value 1 and are indicated on top of each corresponding bar. The data come from two independent experiments and the standard error is represented.

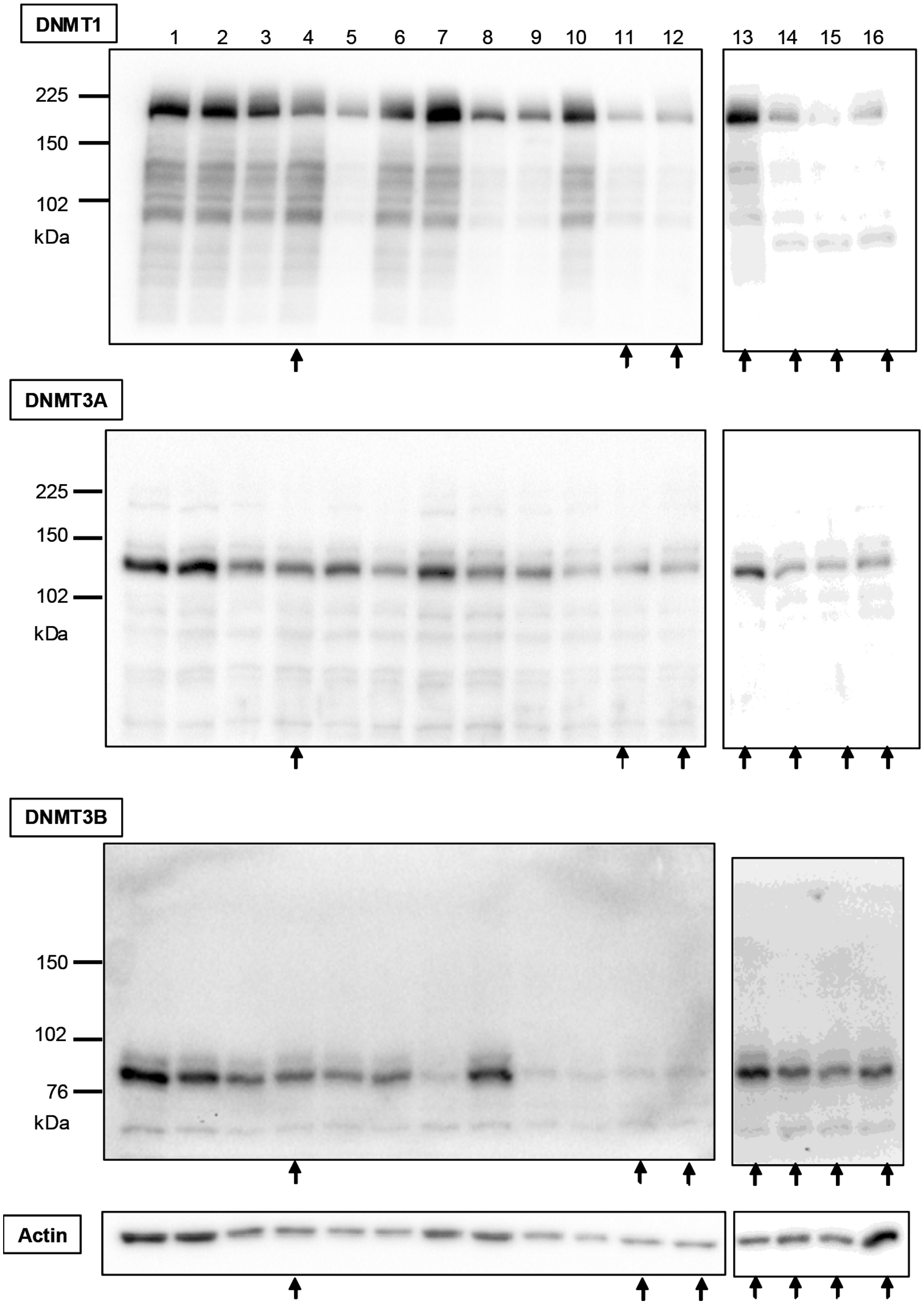

Supplementary Figure S2: Full Western blots corresponding to Figure 1. The arrows indicate the conditions shown in Figure 1. Lane 1: untreated, lane 2: electroporation without siRNA, lane 3: 300 nM of the control luciferase siRNA, lane 4: 700 nm luciferase siRNA, lane 5: *DNMT1(6)* siRNA, lane 6: *DNMT3A* siRNA, lane 7: *DNMT3B* siRNA, lane 8: *DMNT1+3A* siRNA, lane 9: *DNMT1+3B* siRNA, lane 10: *DNMT3A+3B* siRNA, lanes 11 and 12: duplicate of the *DNMT1+3A+3B* siRNA condition. Lanes 13 to 16 corresponds to untreated, 10, 30 and 100 nM DAC treatment.

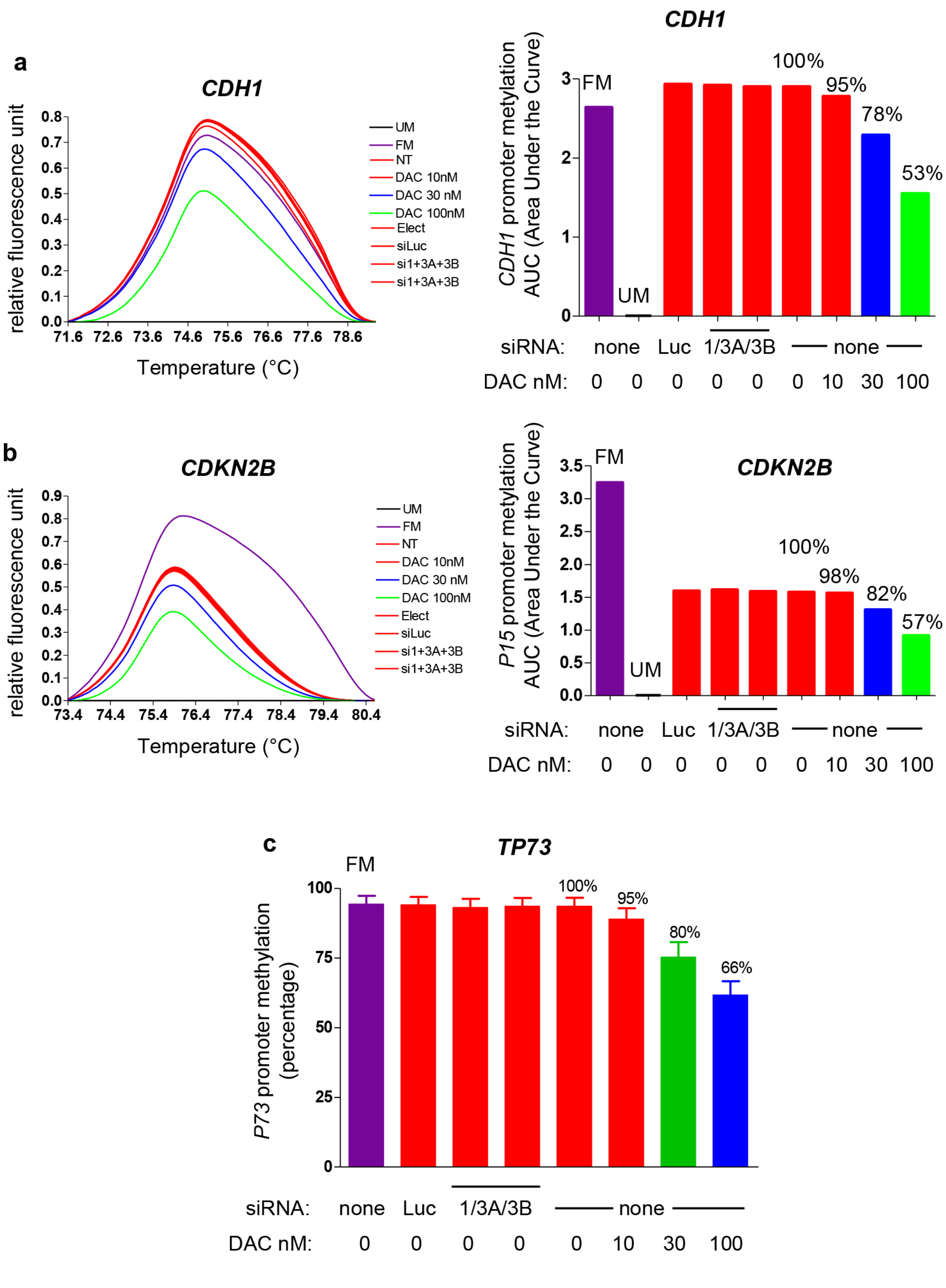

Supplementary Figure S3: Impact of 72 hours depletion of all three *DNMT1/3A/3B* in KG1 cells on promoter methylation of *CDH1* (a), *CDKN2B* (b) and *TP73* (c) genes. MS-HRM was used to measure the methylation level of *CDH1* **(a)** and *CDKN2B* **(b)** promoter regions. For each gene, the raw data showing the substraction from the qPCR amplification curves are shown on the left and the AUCs calculations giving the percentages of methylation are reported as histograms on the right. **(c)** The same bisulfite converted DNA was used to analyze by pyrosequencing the DNA methylation of *TP73* gene promoter. The percentages of methylation indicated on top of the bars correspond to the mean value of methylation for all the CpGs analyzed in the region. The percentages were normalized to 100% for untreated cells. FM, fully methylated DNA control, UM, unmethylated DNA control.

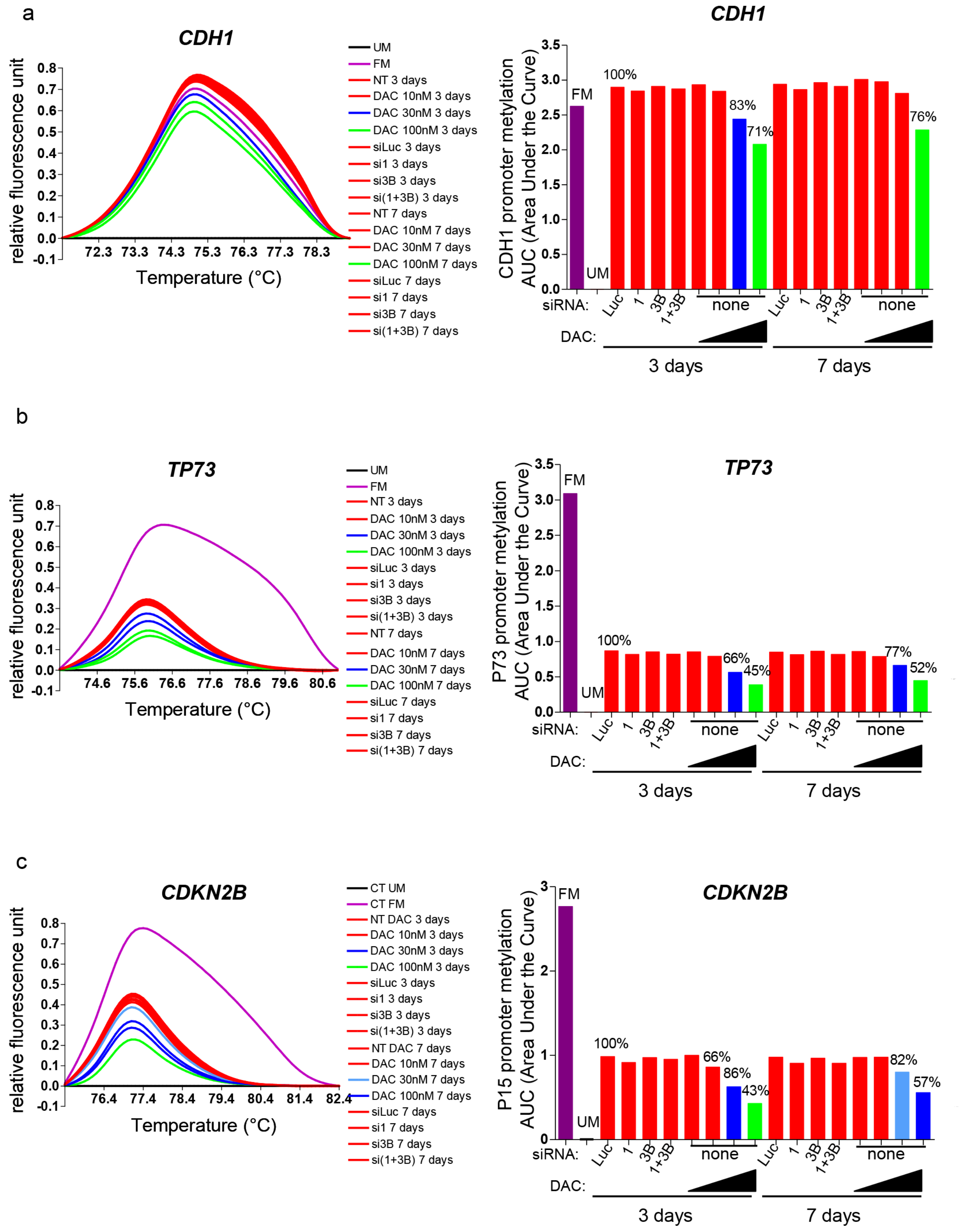

Supplementary Figure S4. DNA methylation variations of (a) *CDH1*; (b) *TP73*; (c) *CDKN2B* measured by MS-HRM after a double transfection of KG1 cells with siRNA directed against *DNMT1* or *3B* separately or combined. For each gene, the raw data and the corresponding histogram with the percentages of methylation are reported. FM, fully methylated DNA control, UM, unmethylated DNA control.

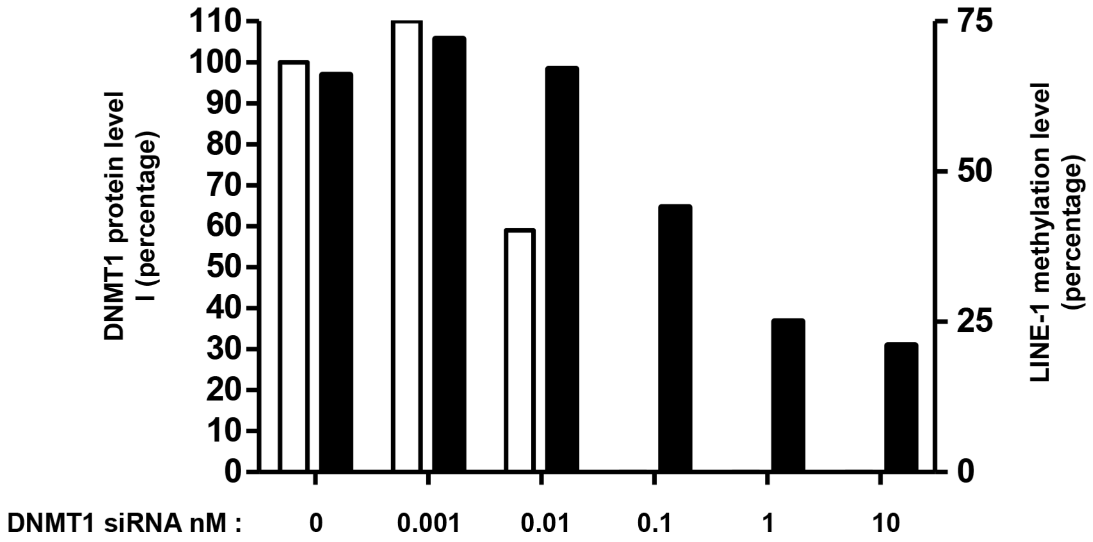

Supplementary Figure S5: Consequences of DNMT1 depletion on *LINE-1* methylation in HCT116 cells. HCT116 were exposed for 72 hours to a dose range of *DNMT1* siRNA (1.6), starting from 0.001 nM up to 10 nM. DNMT1 protein level was determined by Western blotting, while LINE-1 methylation was measured by bisulfite conversion followed by pyrosequencing. White bars, DNMT1 protein level; black bars, LINE-1 methylation level; both measures are expressed as percentage of the control cells exposed to the irrelevant luciferase siRNA used at the same concentrations as the DNMT1 siRNA.

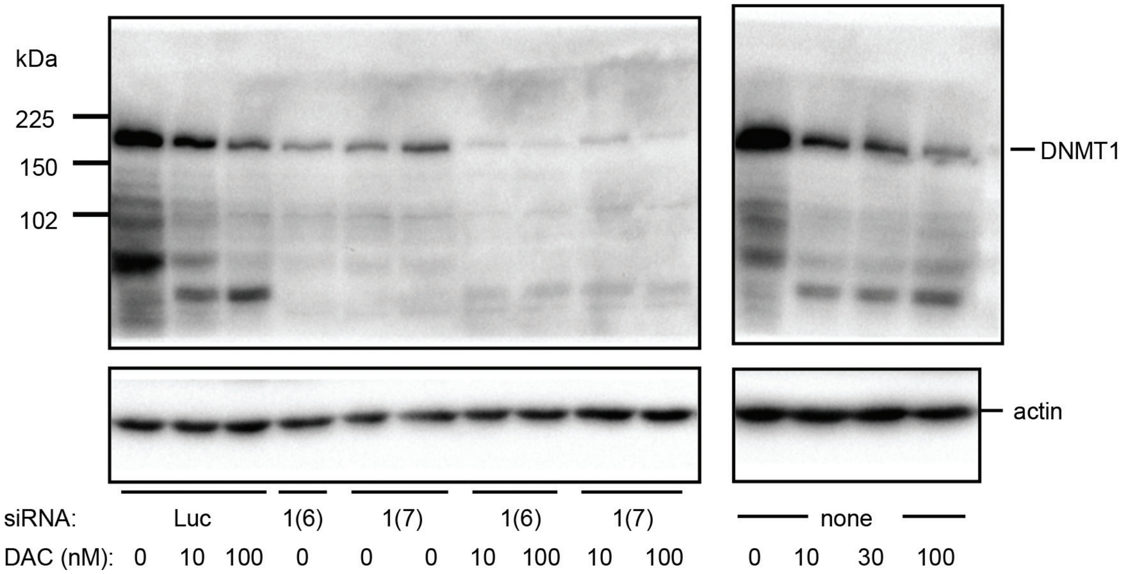

Supplementary Figure S6: Full Western blots corresponding to Figure 3. The protein extracts from cells exposed to the dose range of DAC have been loaded on a separate gel.

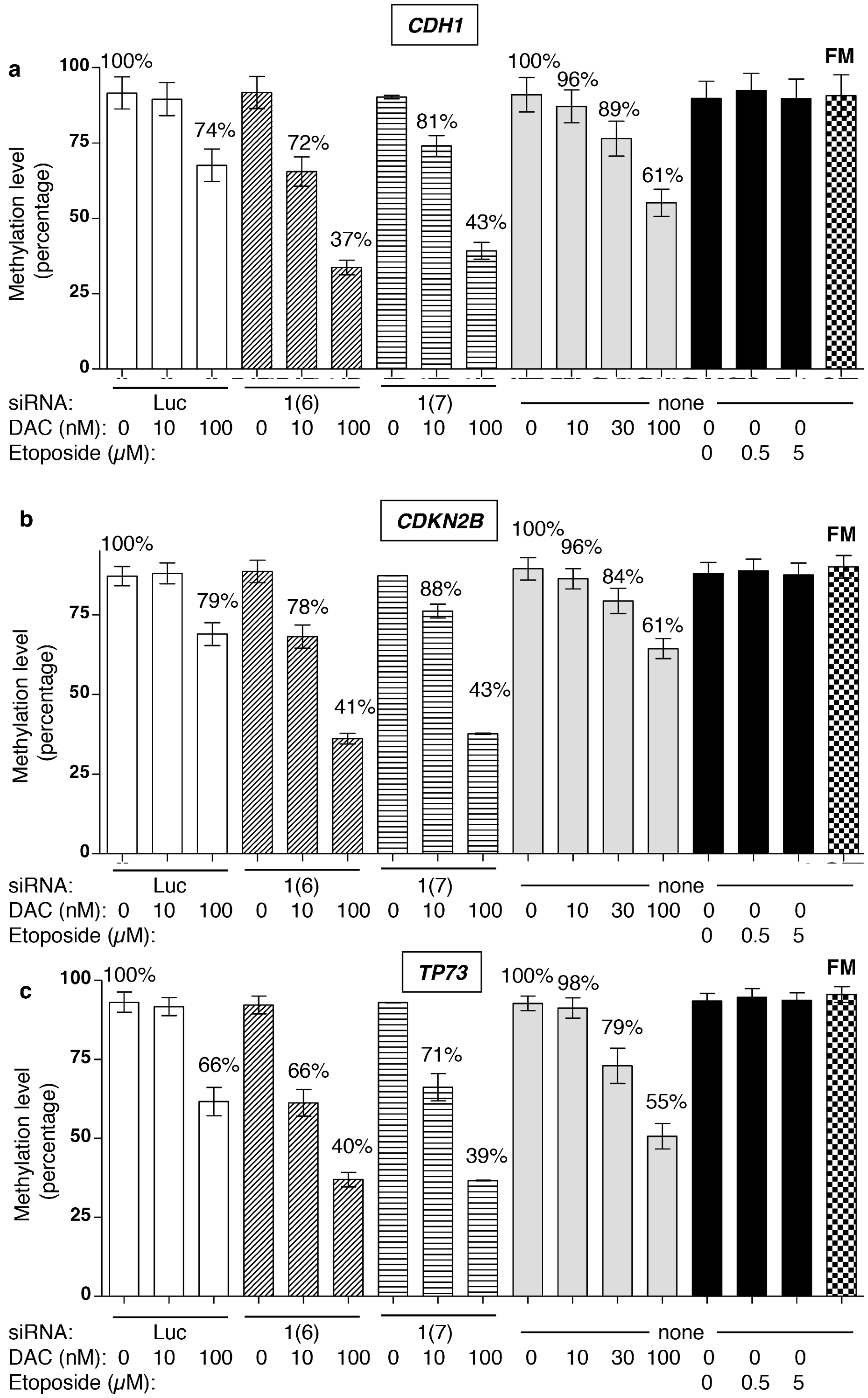

Supplementary Figure S7: Confirmation by pyrosequencing of the MS-HRM analysis on bisulfite converted DNA. KG1 cells were treated for 72 hours with DMNT1 siRNA combined with 10 and 100 nM DAC *versus* DAC and etoposide alone. Genomic DNA was extracted, bisulfite converted and analyzed by pyrosequencing for the methylation level in the promoter regions of *CDH1* **(a),** *CDKN2B* **(b)** and *TP73* **(c)**. The percentage of demethylation (normalized to 100% for the controls, *i.e. luciferase* siRNA and untreated cells) is indicated. FM, fully methylated DNA control.

TP73

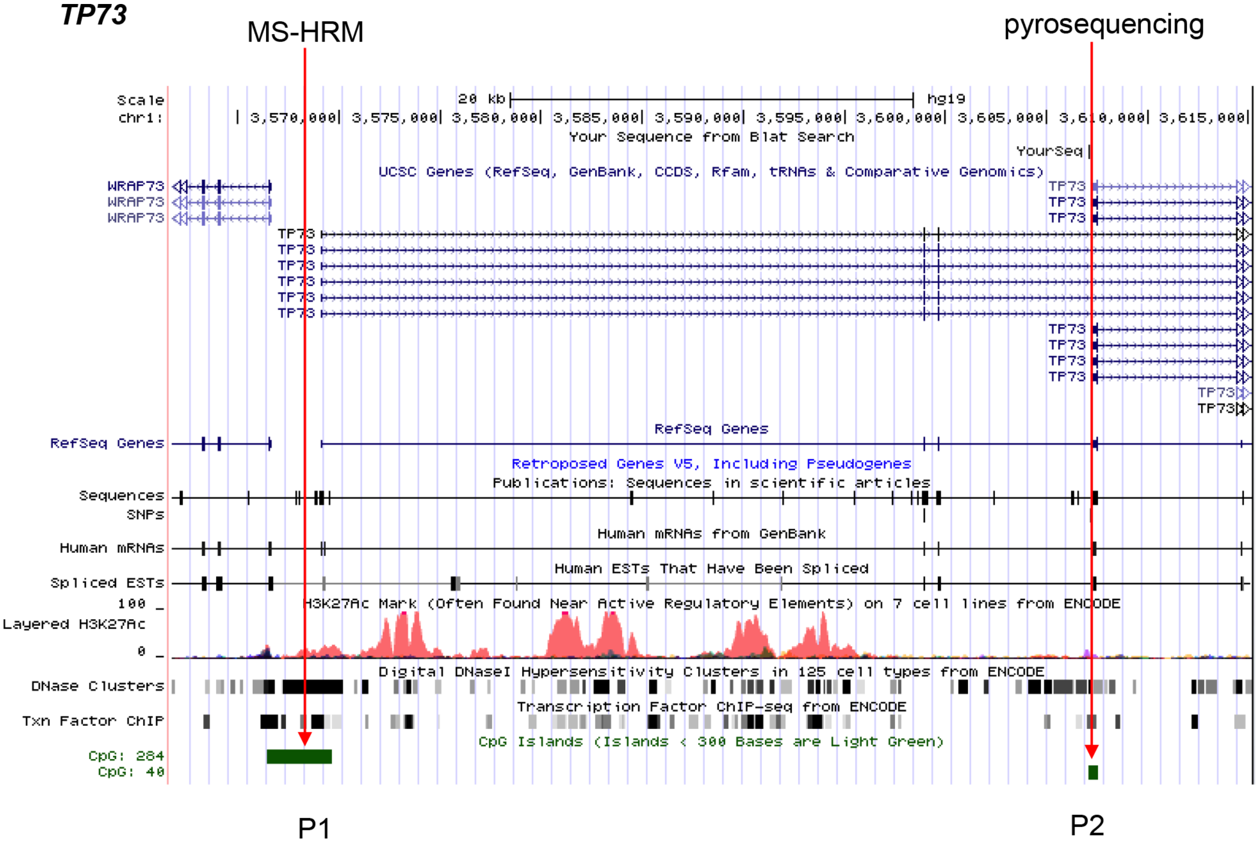

Supplementary Figure S8: Schematic representation of the *TP73* genomic regions analyzed in the MS-HRM and bisulfite conversion-pyrosequencing approaches. P1 and P2 refer to the two promoters described for this gene, source: http://genome.ucsc.edu/index.html

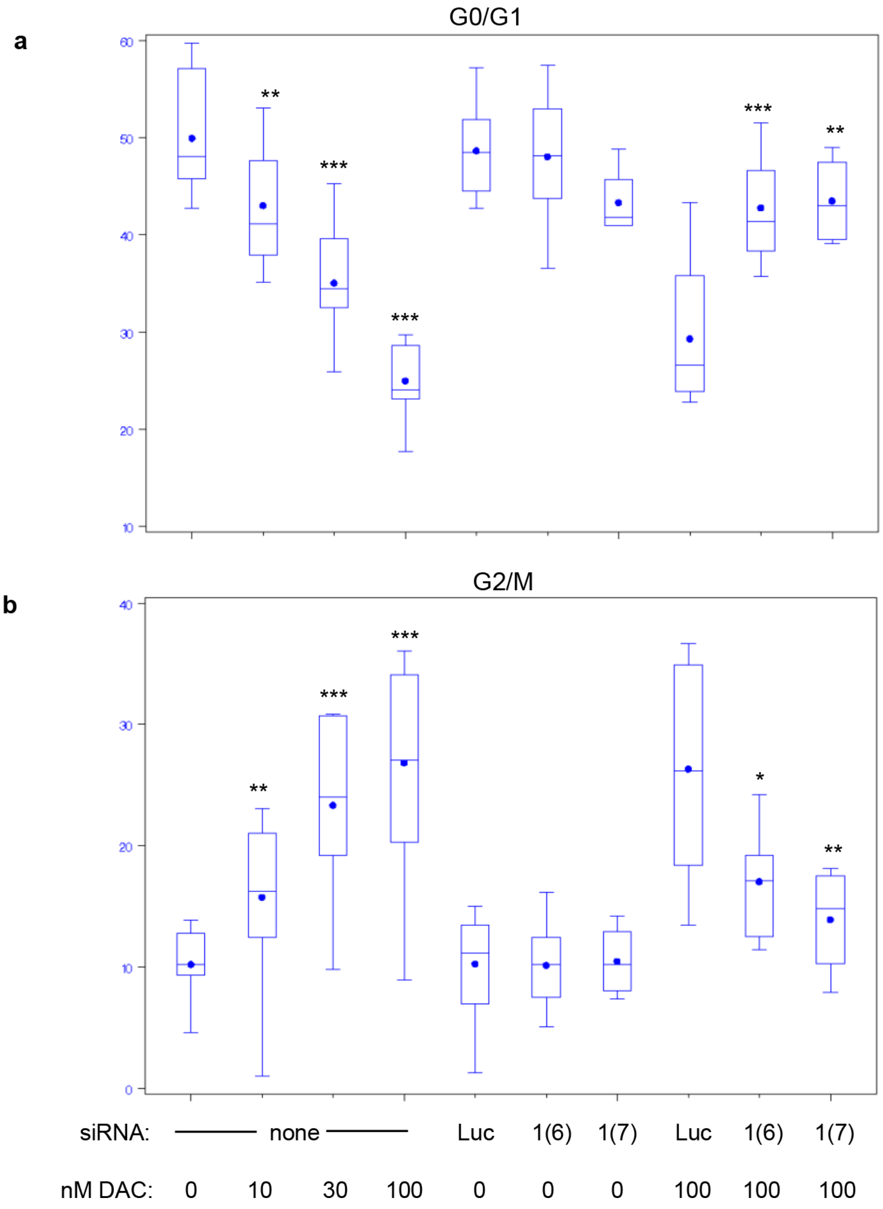

Supplementary Figure S9: *DNMT1* siRNA combined with 100 nM DAC reverts the G2/M blockade induced by DAC. The percentages of cells in the G0/G1 **(a)** and G2/M **(b)** phases are plotted separately in a boxplot representation. KG1 cells treated for 72 hours with *DNMT1* siRNA alone or combined with 100 nM DAC, *versus* DAC alone, as indicated, have been analyzed by flow cytometry for their repartition in G0/G1 and G2/M phases of the cell cycle. The *p*-values represented by one, two and three stars refer to the comparison between the various doses of DAC and the untreated cells and between both *DNMT1* siRNA combined with 100 nM DAC and the luciferase siRNA combined with 100 nM DAC as a control.

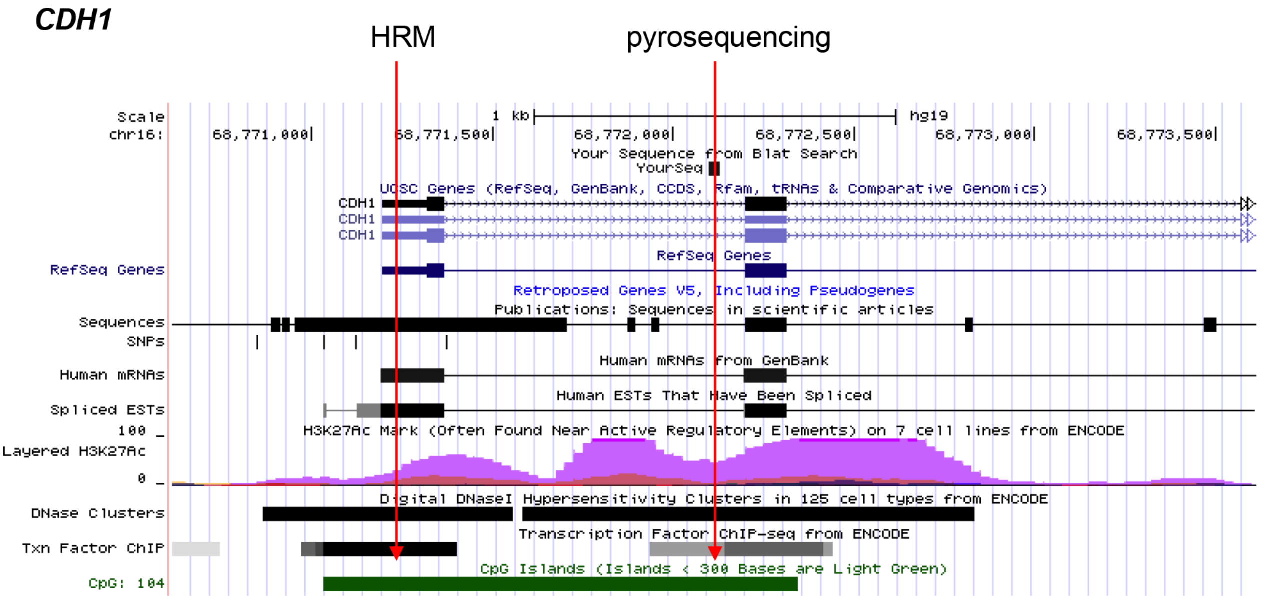

Supplementary Figure S10: Schematic representation of the *CDH1* genomic regions analyzed in the MS-HRM and bisulfite conversion-pyrosequencing approaches. Source: http://genome.ucsc.edu/index.html

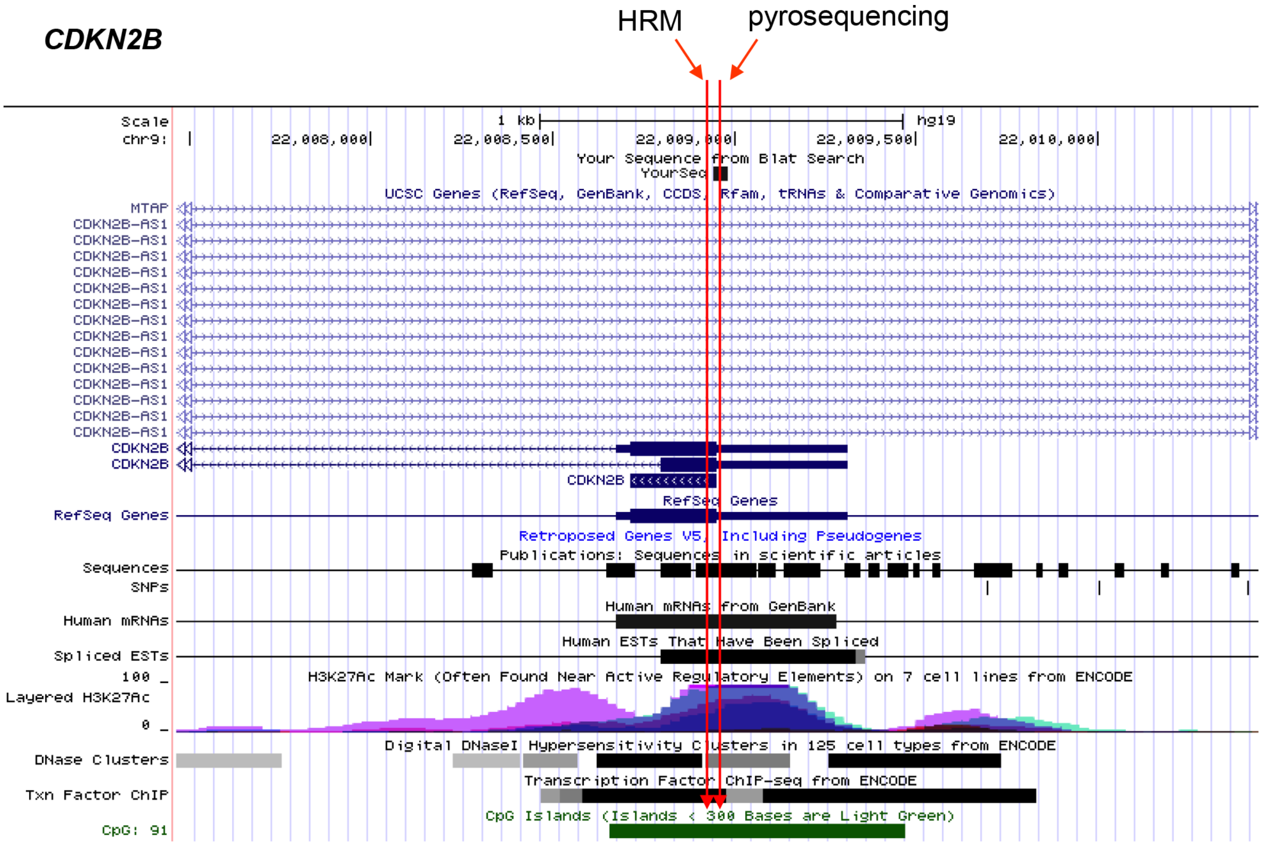

Supplementary Figure S11: Schematic representation of the *CDKN2B* genomic regions analyzed in the MS-HRM and bisulfite conversion-pyrosequencing approaches. Source: http://genome.ucsc.edu/index.html.

Supplementary Table S1: mRNA expression level for various DNMT variants in KG1 and HL60 cells determined by RT-qPCR. Ct refers to the number of qPCR cycles required to pass the threshold (see Materials and Methods). The mean Ct with the standard errors (s.e.) obtained from duplicates are indicated. Several couples of primers were used to amplify all or a fraction of the DNMT variants described (see Materials and Methods).

DNMT	variants	HL60		KG1	
		Mean Ct	s.e.	Mean Ct	s.e.
DNMT1	1 + 2	22.30	0.14	22.55	0.35
DNMT3A	2	35.10	0.14	35.00	0.71
	3	31.65	0.92	30.90	0.14
	1 + 4	33.35	0.35	32.25	0.21
	1 + 2 + 3	28.65	1.06	28.30	0.99
DNMT3B	1 + 2 + 3 + 7 + 8	33.30	N/A	27.50	N/A
	1 + 2 + 3 + 6 + 7 + 8	28.50	0.57	23.55	0.35

Supplementary Table S2: List of genes hypomethylated on at least two CpG in their promoters comparing group 1 *vs*. group 2 (*cfr*. Figure 6 for the definition of these groups)

Supplementary Table S3: List of genes hypermethylated on at least two CpG in their promoters comparing group 1 *vs*. group 2 (*cfr*. Figure 6 for the definition of these groups)
